# Effects of molecular weight and structural conformation of multivalent-based elastin-like polypeptides on tumor accumulation and tissue biodistribution

**DOI:** 10.7150/ntno.39804

**Published:** 2020-01-15

**Authors:** Vijaya Sarangthem, Bo-Yeon Seo, Aena Yi, Young-Jin Lee, Sun-Ha Cheon, Sang Kyoon Kim, Thoudam Debraj Singh, Byung-Heon Lee, Rang-Woon Park

**Affiliations:** 1 Department of Biochemistry and Cell Biology, School of Medicine, and Cell & Matrix Research Institute, Kyungpook National University, Daegu 41944, Republic of Korea.; 2 Department of Pathology, All India Institute of Medical Sciences, New Delhi-110029, India.; 3 Department of Medical Oncology Lab., All India Institute of Medical Sciences, New Delhi-110029, India.; 4 Laboratory Animal Center, Daegu-Gyeongbuk Medical Innovation Foundation, Cheombok, Daegu, 41061, Republic of Korea

**Keywords:** multivalent, size-dependent, tumor targeting, IL-4 receptor, ELP, biodistribution

## Abstract

In order to improve clinical outcomes for novel drug delivery systems, distinct optimization of size, shape, multifunctionality, and site-specificity are of utmost importance. In this study, we designed various multivalent elastin-like polypeptide (ELP)-based tumor-targeting polymers in which multiple copies of IL-4 receptor (IL-4R)-targeting ligand (AP1 peptide) were periodically incorporated into the ELP polymer backbone to enhance the affinity and avidity towards tumor cells expressing high levels of IL-4R. Several ELPs with different molecular sizes and structures ranging from unimer to micelle-forming polymers were evaluated for their tumor accumulation as well as *in vivo* bio-distribution patterns. Different percentages of cell binding and uptake were detected corresponding to polymer size, number of targeting peptides, or unimer versus micelle structure. As compared to low molecular weight polypeptides, high molecular weight AP1-ELP showed superior binding activity with faster entry and efficient processing in the IL-4R-dependent endocytic pathway. In addition, *in vivo* studies revealed that the high molecular weight micelle-forming AP1-ELPs (A86 and A100) displayed better tumor penetration and extensive retention in tumor tissue along with reduced non-specific accumulation in vital organs, when compared to low molecular weight non-micelle forming AP1-ELPs. It is suggested that the superior binding activities shown by A86 and A100 may depend on the multiple presentation of ligands upon transition to a micelle-like structure rather than a larger molecular weight. Thus, this study has significance in elucidating the different patterns underlying unimer and micelle-forming ELP-mediated tumor targeting as well as the *in vivo* biodistribution.

## Introduction

Most anti-cancer drugs used in cancer therapy show minimal effects due to poor penetration into tumor tissue associated with pathological and physiological components of the tumor microenvironment 1. Tumor environments are characterized by abnormal development of vasculature, which leads to overproduction of immature vessels and intertumoral pressure 2, 3. Thus, anti-cancer drugs need to eliminate tumor cells and bypass the tumor vascular network, which involves crossing vessel walls, penetrating the interstitial space, and finally reaching tumor cells while maintaining an abundant concentration. Numerous drug delivery systems and molecular targeted therapies have been developed as an approach to solve the lack of specificity of conventional therapeutic drugs 4. Nanoparticle- or macromolecule-based drug carriers can be linked to the therapeutic agent covalently and target the perivascular tumor region via passive or active targeting strategies 5-8. Excessive leakiness of the tumor vasculature with large pores (cut off size ≥ 200 nm) and poor lymphatic drainage favors selective accumulation of macromolecular drug delivery systems (micelles, liposomes, gold nanoparticles, etc.) in the tumor 9, 10. Covalent attachment and encapsulation with high molecular weight polymers can even solubilize hydrophobic drugs and enlarge the hydrodynamic radii, which can prevent renal filtration and extend the mean residence time of the drug 11-13.

Moreover, studies have shown that molecular weight is a key parameter of the trans-vascular transport of therapeutic drugs. It was demonstrated that tumor vasculature is less selective than normal vasculature due to large pore size, and a 160 KDa molecule is more permeable than a 25 KDa molecule 14. Model analysis using available data showed that molecules larger than 25 KDa tend to have high tumor accumulation 15. As protein molecular size significantly influences biodistribution, precise control of molecular size is necessary for optimum targeting. Thus, a highly flexible system which can provide controllable nature in terms of size, shape, multi-functionality, and site-specificity are necessary to improve the clinical outcomes of novel drug delivery systems. Genetically engineered biopolymers such as collagen-like polymers 16, silk-like polypeptides (SLPs) 17, extended recombinant polypeptide (XTEN) 18, silk-elastin-like polypeptides (SELPs) 19, and elastin-like polypeptide (ELP) 20 provide unparalleled control over sequence and structure at the genetic level. Elastin like polypeptides (ELPs) are extensively used for a wide range of applications including drug delivery and tissue engineering 21. It consists of pentapeptide sequences repeat VPGXG (where X can be any amino acid except proline) derived from mammalian tropoelastin 22. They are thermally responsive biopolymers which undergo a reversible phase transition at certain temperatures referred to as the transition temperature (Tt). Below the Tt, they exist as soluble unimer state but above the Tt, ELP molecules form insoluble coacervate. By substituting the guest residue X with other amino acids and by varying the molecular weight or concentration of the protein, the Tt can be tailored precisely to match the desired purposes 23. These biopolymers can be modified precisely to have defined architectures and functional properties based on incorporation of targeting ligands or specific site for drug conjugation for particular molecular function 24-26. As these polymers are generally synthesized in cells (prokaryotes or eukaryotes) and consist of natural or unnatural amino acids, they are less toxic since they are subjected to specific proteolytic mechanisms that leave relatively inert short peptides or amino acids 27. Fusion of other functional peptides or protein domains along the coding sequences of these polymers maintain its biological activity and allow purification with high yield and low cost amenable to scale-up in biological systems 28, 29. Thus, pharmacokinetic parameters such as biodistribution and clearance rate can be controlled based on the molecular weight, which has an impact on *in vivo* drug carrier disposition 30. Moreover, due to thermal responsive phase behavior, ELP can be tuned to assemble into nanoparticle-like structures in aqueous solution while sustaining the bio-activities of the fusion proteins.

ELP-based nanoparticles have been successfully exploited for the delivery of many clinically approved chemotherapeutics drugs in preclinical model 21. Elastin-like polypeptide conjugated with anti-cancer drugs like doxorubicin or paclitaxel have shown promising therapeutic effects in solid tumor models including glioblastoma, prostrate and breast cancer 31-33. Incorporation of a targeting peptide onto this ELP-chemotherapeutic construct will further improve greater drug uptake by tumor cells without much adverse effect.

In this study, we investigated the tumor-targeting activity of various ELP polypeptides containing IL-4 receptor (IL-4R)-targeting peptides (AP1) based on the size and architecture of nanoparticles in tumor accumulation as well as biodistribution. Previously, we reported that [AP1-V_12_]_6_, ELP-based polymer consisting of six repeats of AP1 increased binding avidity and affinity towards IL-4R. The polymer was highly localized to IL-4R-expressing tumor tissue after intravenous injection and was retained up to 24 h. Moreover, [AP1-V_12_]_6_ showed higher accumulation in the liver and kidney, which may be attributed to its lower molecular weight 34, 35. Thus, various multivalent-based AP1- ELP polypeptides containing random repetitions of IL-4R- targeting peptide with different molecular weights were generated.

The studies have reported that high molecular weight soluble ELPs (more than 70 KDa) were retained in the blood for a long time, resulting in EPR-based enhancement of tumor accumulation in a breast cancer xenograft model, whereas low molecular weight soluble ELP (less than 40 KDa) was cleared rapidly by the kidneys 21. Besides the molecular weight variation, amino acid sequence and nanostructure formation by elastin-like polypeptides (ELPs)-based polymers have great impact on their pharmacokinetic or biodistribution properties in orthotopic breast cancer mice model 36. The micelle forming ELP block copolymer (78 KDa) and a free ELP of similar length (74 KDa) shared the similar pharmacokinetics or tumor accumulation pattern. But long ELPs (74 KDa) displayed higher heart activity with half-life of 8.7 h than short ELPs (37 KDa) which half-life of 2.1 h. Thus, emphasizing the importance of molecular weight in controlling the fate of these polymers. Therefore, the physical characteristics and *in vivo* tumor-targeting efficiency in accordance with the molecular weights were determined. Additionally, we evaluated the different mechanisms underlying unimer and micelle-forming ELP-mediated tumor targeting as well as biodistribution *in vivo*.

## Results and Discussion

### Design and Synthesis of AP1-ELPs

**Figure 1:**
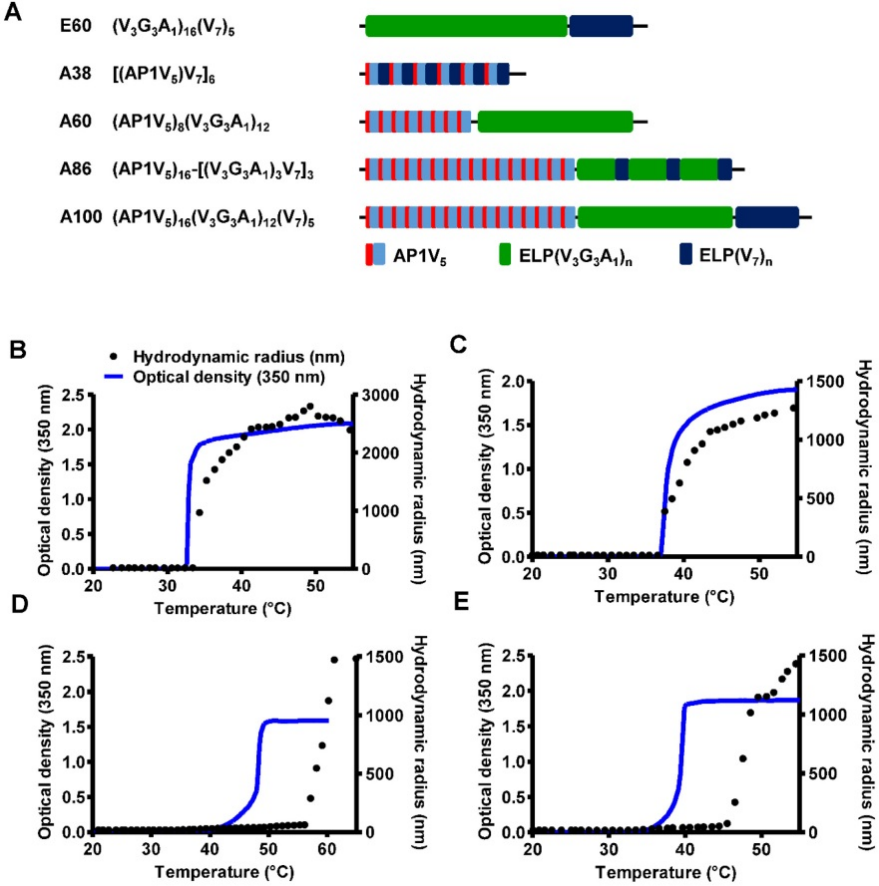
** Characterization of ELP and AP1-ELP polypeptides.** (A) Schematic representation of ELP and AP1-ELPs with corresponding amino acid sequences. Hydrodynamic radius (nm) and turbidity profiles of A38 (B), A60 (C), A86 (D), and A100 (E) were monitored at different temperatures. Data are represented as mean ± SD (n = 3).

The abnormal development of tumor vascularture, and other physio-pathological condition of tumor microenvironment hinder the penetration of drugs into the tumor tissue results in antitumor drug resistance. Thus, a favorable system which can induce site-directed delivery of the drug by penetrating heterogeneous tumor microenvironments are of great importance. Thus, in this study we have generated stimulus responsive, multivalent-based tumor targeting ELPs with variable architecture, molecular weight, physicochemical properties which can self-assemble to form diverse structure and analyzed its clinical outcomes in terms of particle size, shape, multifunctionality, and site directed delivery. As the molecular size greatly affects the fate of ELP polypeptides *in vivo*, we evaluated the behaviors of five ELPs with different molecular sizes. The five polypeptides (E60, A38, A60, A86, and A100) were constructed using the recursive directional ligation (RDL) method. Figure 1A shows the corresponding amino acid sequences of the chimeric polypeptides. Devoid of the IL-4R-targeting peptide, E60 was used as the non-targeting control. For effective blood circulation, all polypeptides were designed to be soluble at the physiological temperature. All polypeptides were successfully expressed and purified with a yield of ~30 mg/L using the inverse transition cycling (ITC) method. The size and purity of these polypeptides were confirmed by SDS-PAGE, followed by copper chloride staining (Figure S1, Supporting Information). A faint extra band with a size double that of the expected proteins was observed in SDS-PAGE, which could be attributed to dimer formation caused by the presence of Cys residues at the C-terminus. Determination of the exact molecular weight by MALDI-TOF/TOF MS confirmed the protein sizes of E60 (59438.98 Da), A38 (38170.22 Da), A60 (59846.40 Da), A86 (86261.07 Da), and A100 (100485.50 Da) respectively (Figure S2A and S2B, Supporting Information).

### Thermal Properties and Particle Size Determination

Thermal transition of the samples were characterized by measuring the optical density (OD) at 350 nm using a UV-visible spectrophotometer at a rate of 1°C/min. Turbidity profiles clearly indicated that A38, A60, and E60 exhibit a single transition from the soluble unimer to the insoluble aggregate form, as shown by dramatic increases in OD (Figure 1B, 1C and Figure S3, Supporting Information). The transition temperatures (Tt; 50% of maximum aggregation) were within the range of 38-40°C, just above the physiological temperature. On the other hand, A86 and A100 showed three-step transitions such as unimer-micelle-aggregate (Figure 1D, 1E), consistent with ELP block polymers (ELP_BC_) 37, 38. At a lower temperature, the samples remained as soluble unimers (OD _350_<0.1). The critical micelle temperature (CMT) at which the unimer-to-micelle transition occurred was reflected by a subsequent increase in the absorbance (OD_ 350_∼0.1-0.8) with increase in the temperature (Figure S4, Supporting Information). A further rise in the temperature over its CMT resulted in large micron-sized aggregate formations (OD_ 350_∼1.2-1.8).

Determination of particle size by dynamic light scattering (DLS) demonstrated a three-step transition behavior consistent with the turbidity profiles. In lower temperature below 35.4 °C, A86 occurred as a stable unimer with the hydrodynamic radius (Rh) of 18.7-19.7 nm. The increased in the absorbance (∼0.1 - 0.8 OD_350_) indicating the nanoparticle formation was observed within the CMT range of 35.4 - 56.4 °C, corresponds with slight increase of hydrodynamic radius (Rh) range of 21.17 - 62.4 nm due to partial transition of the ELP block with low transition temperature. Further increase in the temperature above CMT (~57.7 - 64.9 °C), triggered inverse temperature phase transition of A86 which induced the formation of large micrometer-sized aggregates (287.5 - 1481.3 nm) (Figure 1D and Figure S4, Supporting Information). Likewise, A100 occurred as the unimer, with Rh of ~19.14 nm (below 33°C), and transition into a micelle, with Rh of 20.2 - 76.01 nm, at 33.5 - 45.5 °C respectively. The micelle- aggregate transition was observed at ∼46.5 - 56.6 °C (255 - 1407.4 nm) (Figure 1E and Figure S4, Supporting Information).

At physiological temperature, A86 and A100 self-assembled to form nanoparticles with a size of ~25.54±6.4 nm or ~39.3±3.29 nm respectively, (Figure 1D, 1E) while A60 had a particle size of ~388±19 nm (Figure 1C). In addition, E60 and A38 had the particle sizes of ~1703 nm and ~1880 nm, respectively, which were relatively larger compared to other polypeptides (Figure 1B and Figure S3, Supporting information). Further increase in the temperature led to micron-sized aggregate formation in all cases. Retention of nanoparticle-like structures by A86 and A100 up to an extended range of temperatures indicated that both the polypeptides remained in a nanoparticle formulation upon systemic administration, demonstrating no obstruction of blood vessels.

Stability of nanoparticle formation by A86 and A100 were further confirmed using DLS after 24 h incubation in 100% mouse plasma at 37°C. An insignificant increased of self-assembled nanoparticle formation by A86 and A100 clearly revealed that both polypeptides can form stable self-assembled micelle like structure in the plasma (Figure S5, Supporting Information). Likewise, no significant change in the hydrodynamic radii of E60, A38 and A60 were observed after 24 h incubation in the plasma which ensure the stability of polypeptides for longer time in physiological condition. SDS-PAGE analysis further confirmed the stability of these polypeptides in the plasma after 24 h (Figure S6, Supporting Information).

**Figure 2:**
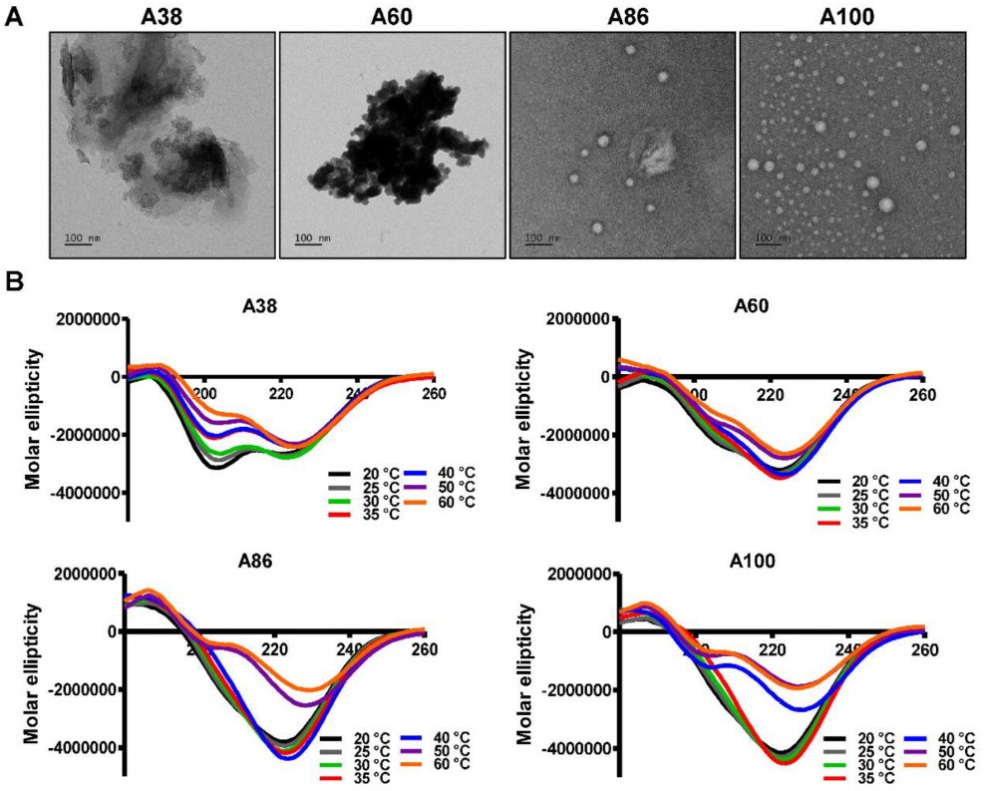
** Structural determination of AP1-ELPs.** (A) TEM images of A38, A60, A86, and A100 at 37 °C, scale bars, 100 nm. (B) Circular dichroism spectra of polypeptides were taken at different temperatures (20, 25, 30, 35, 40, 50, and 60 °C). Data are represented as mean ± SD (n = 3).

To directly visualize the structure, size, and shape of the polypeptides, Cryo-TEM images were taken after incubating the proteins at 37°C. The TEM images clearly showed that A86 and A100 retained their spherical micelle-like structures with particle diameter sizes of 38~40 nm and 42~50 nm (Figure 2A), respectively. On the other hand, A38 and A60 remained in the aggregate form having large micron sizes, clearly confirming a one-step transition consistent with the turbidity profiles and the DLS results. Similarly, E60 formed large-sized aggregates due to higher hydrophobicity and lower the Tt (Figure S7A, Supporting Information).

### Secondary Structure Characterization

Changes in the secondary structure (helix, beta, turn, and random) in response to temperature were monitored using circular dichroism (CD). We investigated whether the thermal transition and different forms of the designed polypeptides correlated with the conformational changes. The CD spectra of E60 acquired as a function of temperature was consistent with ELPs of different length and guest residue compositions reported in other studies 39-42. CD spectroscopy of E60 revealed helix formation, which was reflected by negative bands at 208 nm, 222 nm and positive band at 190 nm (Figure S7B, Supporting Information). On the other hand, A38 and A60 showed a gradual deeper negative peak at 222 nm compared to E60 with subsequent increased in temperature which clearly indicates a helix conformation (Figure 2B). Increased helix formation may be attributed to the presence of multiple copies of hydrophilic-targeting ligands (AP1) along the ELP backbone. The negative peak of A60, A86 and A100 were profoundly deeper than that of A38, which may be directly correlated with the greater number of targeting peptides. The spectra of all the polypeptides were characterized by high percentage of random coils at lower temperature of 20 °C (Table S1, Supporting Information). The consistent gradual structural shift from disordered random coiled structure to highly ordered β-turn and β-sheet at higher temperatures by E60 correspond to the structural shift between the soluble monomers to the insoluble aggregates with increasing temperature 42, 43. Likewise, the targeted polypeptides A38 and A60 displayed a gradual decrease in random coil corresponding to a simultaneous constant increase in the β-turn structure conformation. The steady increase in β-turn formation caused by A38 or A60 at higher temperature correlated with an increased turbidity and particle size. Estimation of the mean secondary structure contents revealed that the appearance of β-sheet at 50°C was consistent with an increase in turbidity by A86. At 60°C, we observed a predominant existence of β-sheet conformation with stable aggregate formation (Table S1, Supporting Information). Likewise, β-sheet conformations of A100 appeared at 40°C with predominance at 60 °C, accompanied with increasing turbidity from 39.67°C. Thus, we interpreted that the appearance of ordered β-sheet conformations in response to temperature correlated with increased turbidity as well as large micron-sized aggregate formations.^43^ Similar to other studies, our study further confirmed that the variation in CD spectra occurred in response to temperature and directly correlated with increased turbidity 44, 45.

### Estimation of Cell binding and Uptake

**Figure 3:**
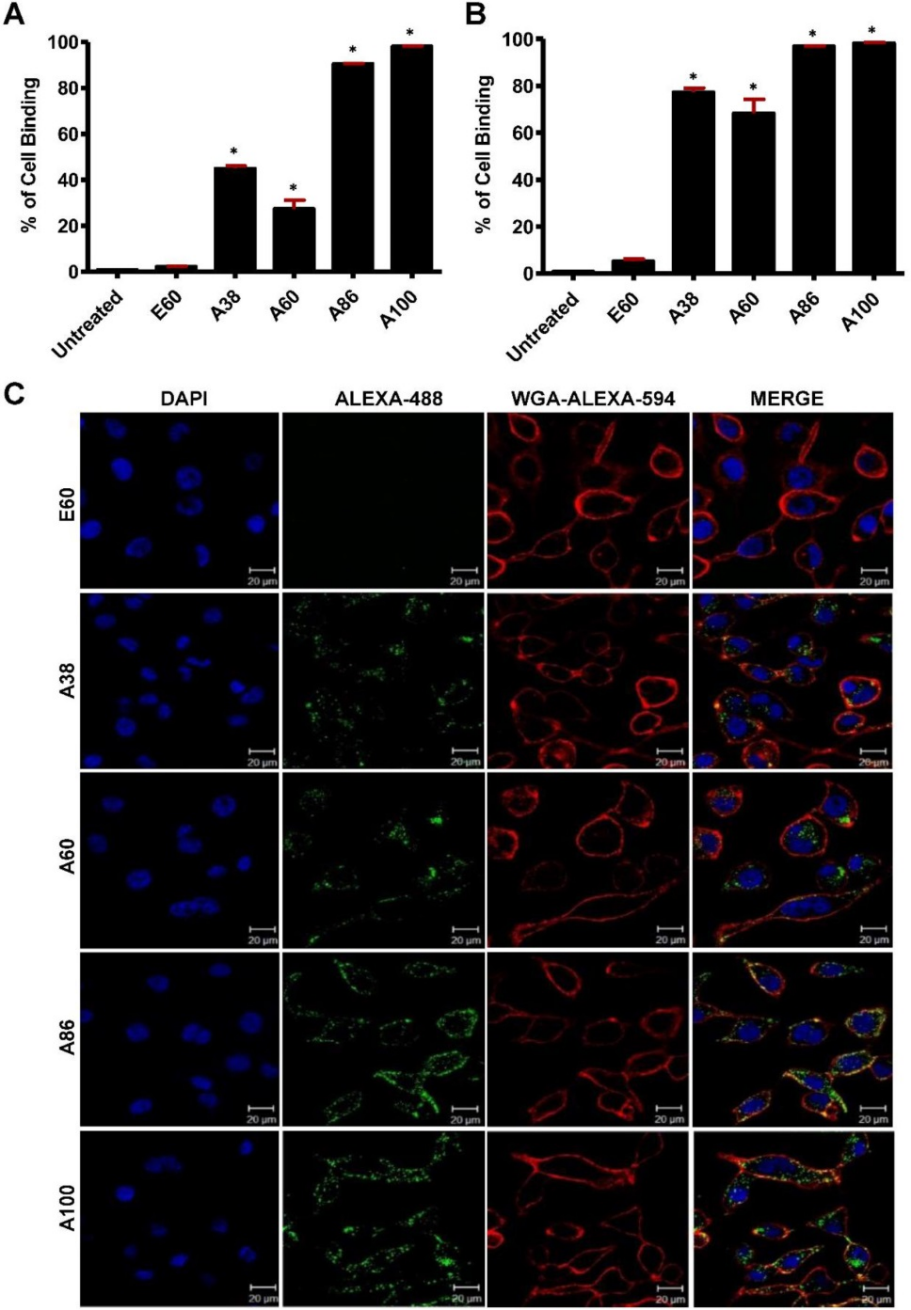
** Determination of cellular binding and uptake.** Histogram represents the percentage of cellular accumulation and uptake determined by flow cytometry after MDA MB231 cells were incubated with fluorescent labeled E60, A38, A60, A86, or A100 at 4°C (A) and 37°C (B) for 1 h. Data are represented as the mean ± SD and analyzed using one-way ANOVA followed by a Tukey post-hoc test, *P<0.05. (C) MDA MB231 cells were treated with 0.3125 *µ*M of the respective polypeptides labeled with Alexa-488 at 37°C for 1 h, and observed under confocal laser microscopy. Cell membrane and nuclei were stained with WGA Alexa 594 and Hoechst, respectively. Representative confocal images of five independent experiments. Scale bar, 20 µm.

To investigate the *in vitro* cell binding efficacy, respective polypeptides were labeled with Alexa-488 at the C-terminal Cys residue. IL-4R-dependent targeting activity was confirmed in 4T1 murine breast cancer and MDA MB231 human breast cancer cells using the flow cytometry, after treatment for 1 h at 4°C. All the targeting polypeptides (i.e., AP1-ELPs) displayed higher cell binding capacity than the non-targeting control E60 in both the cell lines. The cell binding activities of A38 and A60 were 44.9% and 27.3% in MDA MB231 cells, respectively, whereas non-targeted E60 showed minimal binding of 2.2% (Figure 3A). These results clearly indicated that ELP itself did not interact with cells, but instead acts as a support for multivalent presentation of targeting ligands. A38 showed relatively higher binding than A60, indicating that presence of greater number of targeting peptides in the polymer backbone with increased molecular weight had no effect on percentage of binding. The micelle-forming AP1-ELPs (i.e., A86 and A100) displayed superior binding capacities of 90.5% and 98.1%, respectively, which is approximately 2.01±0.13 and 3.3±0.27-fold higher than the non-micelle forming AP1-ELPs (A38 and A60). The evaluation at 37°C in MDA MB231 cells revealed a higher cellular uptake of the targeted AP1-ELPs such as A38, A60, A86, and A100, with the percentages of 77.3, 68.3, 96.9, and 98.2%, respectively (Figure 3B). No significant difference was observed in the uptake by MDA MB231 cells treated with targeting polypeptides, except for A60 showing a nearly 1.13±0.30-fold decreased when compared to A38, A86, and A100. Likewise, A86 and A100 showed relatively higher binding activities of 16 and 42.4% in 4T1 cells respectively (Figure S8A, Supporting Information) compared to other polypeptides. Further analysis of the cellular uptake by 4T1 cells at 37°C revealed maximum internalization values of 32.3 and 58.16% by A86 and A100, respectively, compared to non-targeting control or non-micelle forming AP1-ELPs (Figure S8B, Supporting Information). Differences in the cellular binding and uptake in MDA MB231 and 4T1 cells were solely attributed to the level of IL-4R expression (data not shown). An increase in the molecular weight, along with the targeting peptides of the non-micelle forming AP1-ELPs (A38, A60), did not increase the binding activity, while micelle-forming AP1-ELPs (A86, A100) showed maximum accumulation in tumor cells. Thus, the maximum uptake displayed by micelle-forming A86 and A100 could be attributed to a temperature-dependent phase transition, which possibly mediates micelle formation along with multivalent display of the targeting ligands on the exterior enhancing the cellular localization. On the other hand, E60 showed minimum uptake, clearly demonstrating that internalization of targeting polypeptides was highly dependent on IL-4R-mediated endocytosis and was not the non-specific effect of hyperthermia.

### Mechanism of Cellular Internalization

**Figure 4:**
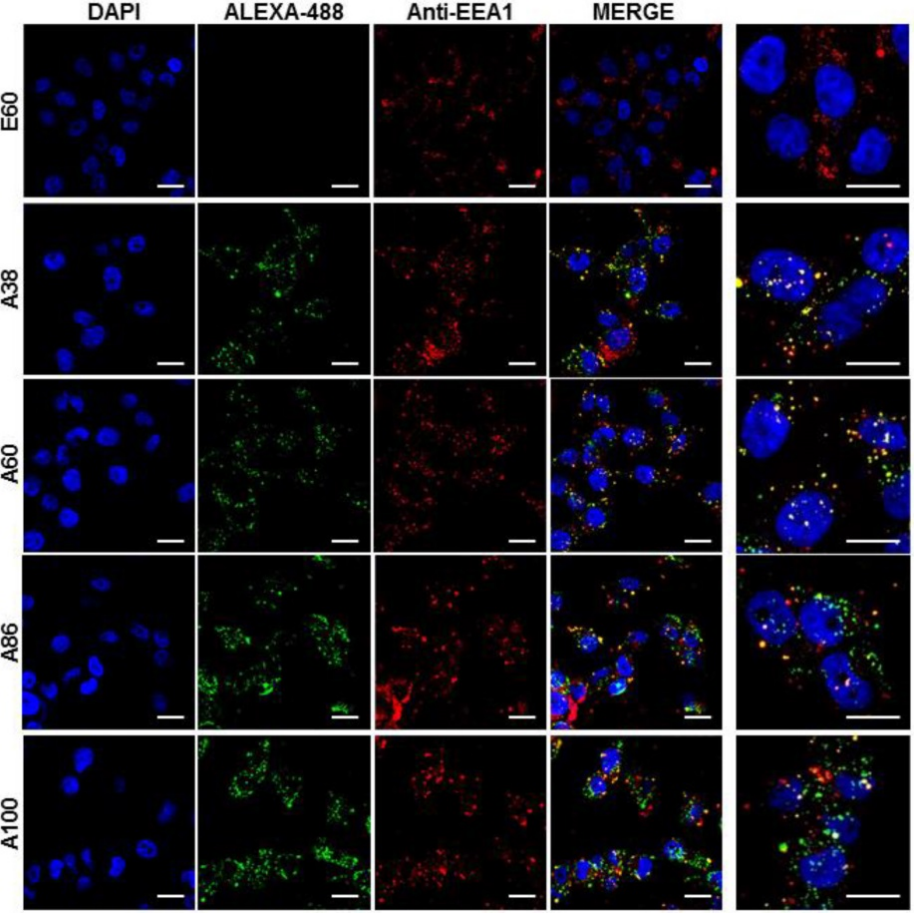
** Examination of subcellular localization.** MDA MB231 cells were incubated with 0.3125 *µ*M of the respective polypeptides labeled with Alexa-488 at 37°C for 30 min and co-localization of polypeptides with early endosomes were observed under confocal laser microscopy. Scale bar, 20 μm*. Right panels*: Examination of co-localization of polypeptides with early endosomes by Z-section scanning of confocal microscopic images. Hoechst: nuclear stain, blue; Red: early endosomes (EEA1); Green: polypeptides labeled with Alexa-488. Representative confocal images of 3 independent experiments. Scale bar, 10 µm.

**Figure 5:**
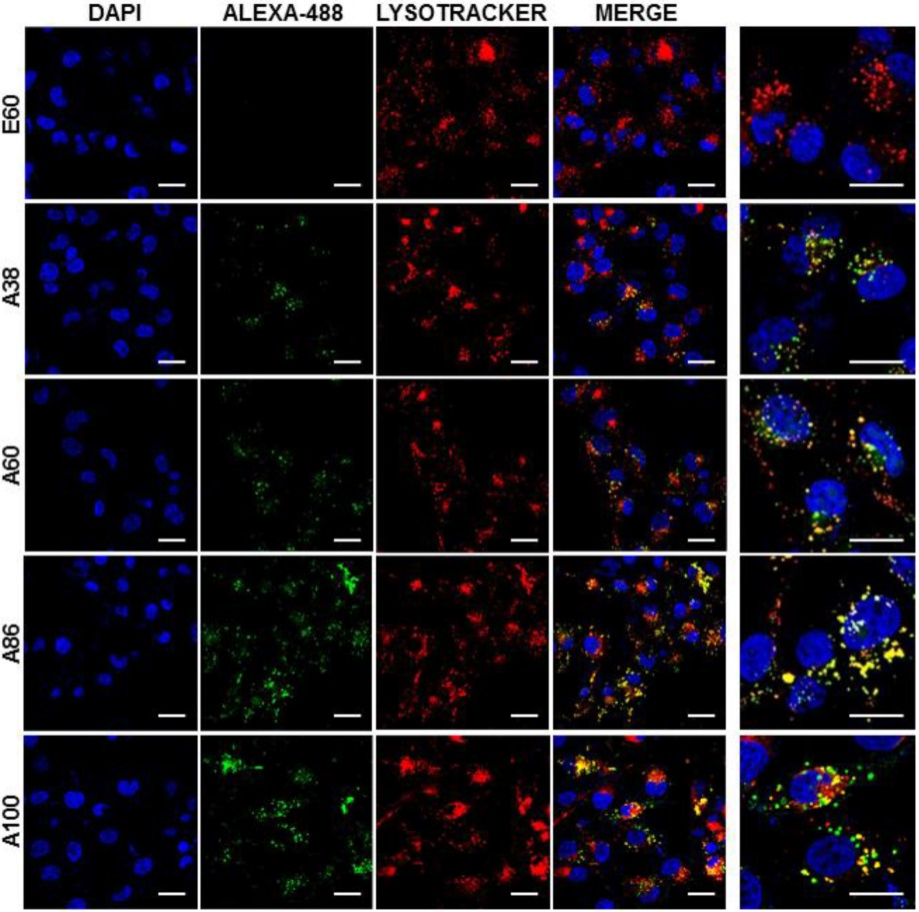
Confocal laser scanning microscopic images of MDA MB231 cancer cells treated with 0.3125 µM of respective polypeptides at 37°C for 1 h. Scale bar, 20 μm. *Right panels*: Examination of co-localization of polypeptides with lysosomes by Z-section scanning of confocal microscopic images. Hoechst: nuclear stain, blue; Red: lysotracker; Green: polypeptides labeled with Alexa-488. Representative confocal images of three experiments Scale bar, 10 μm.

Cell binding activity and cellular uptake of AP1-ELPs were further examined by confocal microscopy. Consistent with the flow cytometry results, all targeting polypeptides displayed efficient cellular localization and uptake compared to non-targeting E60, in both 4T1 and MDA MB231 cells. Interestingly, micelle-forming A86 and A100 polypeptides showed maximum accumulation and uptake in MDA MB231 cells compared to other polypeptides (Figure 3C and Figure S9, Supporting Information). There was no significant difference in cellular accumulation between A38 and A60, which suggested that an increase in number of targeting peptide was not the key factor for the optimal binding. Moreover, an increase in the molecular weight in the case of non-micelle forming polypeptides such as A60 resulted in comparatively less accumulation in cells, which may be attributed to minimal exposure of the targeting ligands. Together, these results suggested that the extreme uptake shown by A86 and A100 may be due to micelle formation, which might reduce the polymer size as well as increase the exposure of targeting peptides on the exterior, thereby allowing more effective internalization. Similarly, A86 and A100 showed maximum accumulation and uptake compared to non-micelle forming polypeptides (A38 and A60) in 4T1 cells (Figure S10 and S11, Supporting Information). The molecular weight dependency in cellular internalization were not clearly visible under *in vitro* conditions; thus, it was expected that the individualized targeting activity displayed by respective polypeptides could be due to their structure transition in response to the temperature or the valance of the targeting ligands constitution upon transition. We further examined the subcellular localization patterns of the polypeptides and checked whether their internalization patterns were truly based on ligand-dependent endocytosis or on the non-specific effect of hyperthermia. In addition, we also monitored the size-dependent subcellular localization, since cellular uptake and efficiency of processing in the endocytic pathway are dependent on particle size 46. Thus, co-localization of the labeled polypeptides in distinct subcellular locations such as early endosomes (EEA1) and lysosomes (lysotracker) were monitored. The anti-EEA1 staining after 30 min of incubation with the polypeptides (Figure 4) showed overlapping of red dots (EEA1) with green (AP1-ELPs), suggesting rapid localization to early endosomes, that subsequently disappeared after 1 h (Figure S12, Supporting information). On the other hand, yellow spots were observed resulting from overlapping of the green with the red signal for lysosomes, suggesting polypeptide localization at lysosomes after 1 h (Figure 5). Thus, after binding with IL-4R, all the polypeptides underwent a receptor-mediated endocytic mechanism, which was denoted by localization to early endosomes followed by movement to lysosomes. A38 and A60 revealed maximum co-localization with early endosomes and lesser localization to the lysosomes, whereas A86 and A100 showed maximum localization with lysotracker in a short time period corresponding to minimal overlapping with early endosomes, clearly indicating faster entry of polypeptides compared to non-micelle forming polypeptides. However, no uptake and co-localization was observed in the cells incubated with E60. These results suggested that the superior binding activities shown by A86 and A100 may depend on multiple presentation of ligands upon transition to a micelle-like structure, rather than an increased number of targeting ligands.

### *In vivo* Biodistribution of AP1-ELPs

To examine the pharmacokinetic properties, biodistribution, and tumor-targeting activities of AP1-ELPs *in vivo*, FPR 675-labeled polypeptides (100 µM) were injected intravenously into the 4T1 tumor xenograft Balb/C wild-type mice. In this study, the 4T1 tumor xenograft mouse model was used due to its property of rapid cell proliferation with the subsequent forming of subcutaneous solid tumors within a short period. Determination of pharmacokinetic properties after intravenous administration revealed that all AP1-ELPs polypeptides were readily cleared from the circulation (Table S2). The plasma half-life of A38 was 60.7±0.31 h, which increased with the higher molecular weight A60 (68.6±0.57 h). Thus, we observed that the increased molecular weight resulted in the increased retention in the blood circulation with maximum exposure to tumor tissue 36, 47. All the targeting polypeptides A38, A60, A86 and A100 displayed maximum half-lifes (60.7 h, 68.6 h, 64.5 h and 63.6 h respectively) than non-targeted E60 (25.5 h). So, it could be interpreted that functionalization of ELP with tumor specific ligands facilitated prolong circulation of the polypeptides in the blood stream. Moreover, A60 exhibited slightly better half-life than A86 and A100. Thus, these results suggested that besides increased in the molecular weight of polypeptides, their structural conformation and size play a vital role in determining the fate of polymer 48.

**Figure 6:**
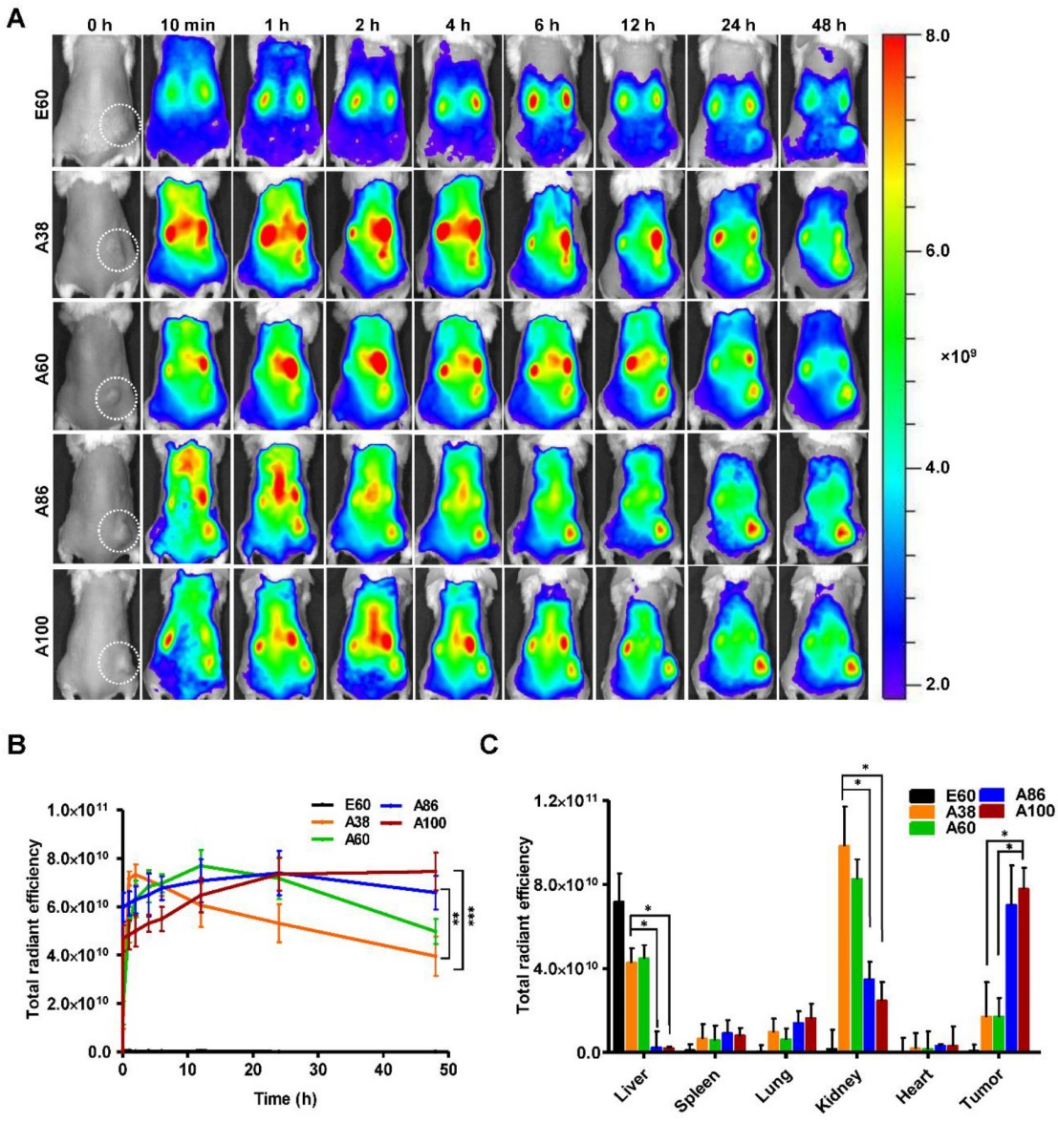
*** In vivo* biodistribution imaging of AP1-ELPs in allograft mice model.** (A) FPR 675-labeled E60, A38, A60, A86, and A100 (100 *µ*M) were injected intravenously into 4T1 tumor allograft mice. The *in vivo* fluorescence images were collected at different time points (10 min, 1, 2, 4, 6, 12, 24, and 48 h) after intravenous injection (IV) to determine biodistribution and a representative image of each group is shown. (B) Quantification of fluorescence intensities in tumor sites at different time intervals. All data are presented as mean ± SD (n = 6), statistical significance was determined by one-way ANOVA analysis, (***P<0.001, **P<0.01). (C) Fluorescence intensities of excised tumors and organs at 48 h post-injection (n = 10). Data are represented as mean ± SD and analyzed using one-way ANOVA, (n=3), *P<0.05.

*In vivo* fluorescence images taken by an IVIS *in vivo* imaging system at different time points (10 min, 1, 2, 4, 6, 12, 24, and 48 h) displayed different patterns of time-dependent tumor accumulation and biodistribution, and were found to be dependent on the molecular weight (Figure 6A). Non-targeting E60 was observed to accumulate non-specifically in off-target tissues. Low molecular weight A38 rapidly accumulated as early as 1 h, reaching maximum intensity at 2 h, followed by a steady decrease over time. In addition, A60 showed a steady increase at tumor sites in a time-dependent manner, with maximum accumulation at 12 h, which then reduced over 48 h (Figure 6B). These data clearly demonstrated that an increase in the molecular weight resulted in longer retention at the tumor sites as well as less non-specific accumulation. Furthermore, A86 showed higher accumulation at 10 min post-injection and persisted until 48 h. This mode of accumulation displayed by A86 was likely due to its micelle-like formation upon injection rather than an increase in molecular weight. Likewise, A100 showed a similar pattern of accumulation in tumors, as evidenced by a gradual increase in the fluorescent intensity that was maintained up to 48 h. Comparatively, A86 and A100 showed minimum off-target tissue accumulation along with relatively reduced accumulation in the kidneys, followed by rapid degradation over time.

*Ex vivo* fluorescence images of tumors and organs isolated at 48 h post-injection from 4T1 allograft mice further verified that most AP1-ELPs were intensely accumulated in tumors, except for E60. Consistent with *in vivo* imaging results, the *ex vivo* images showed similar tumor accumulation patterns. The low molecular weight A38 showed a weaker signal in tumor tissue after 48 h, with persistent increases in the liver and kidneys due to rapid degradation (Figure S13C, Supporting Information). The higher molecular weight A60 showed relatively reduced accumulation in the kidneys, whereas there was no significant change in liver accumulation. In addition, A86 and A100 displayed maximum tumor accumulation and longer retention compared to other polypeptides. Fluorescence intensities in the targeted tumors increased up to 3.5± 0.07 and 3.8± 0.85 times with A86 and A100, respectively, as compared with A38 (Figure 6C). Gradual increases in the molecular weight and the number of targeting peptides considerably reduced liver accumulation. For kidneys, 1.2 and 2.45-fold reductions by A86 and A100, respectively, were detected in comparison with A38. All the polypeptides showed negligible accumulation in other organs such as the spleen, lungs, and heart.

**Figure 7:**
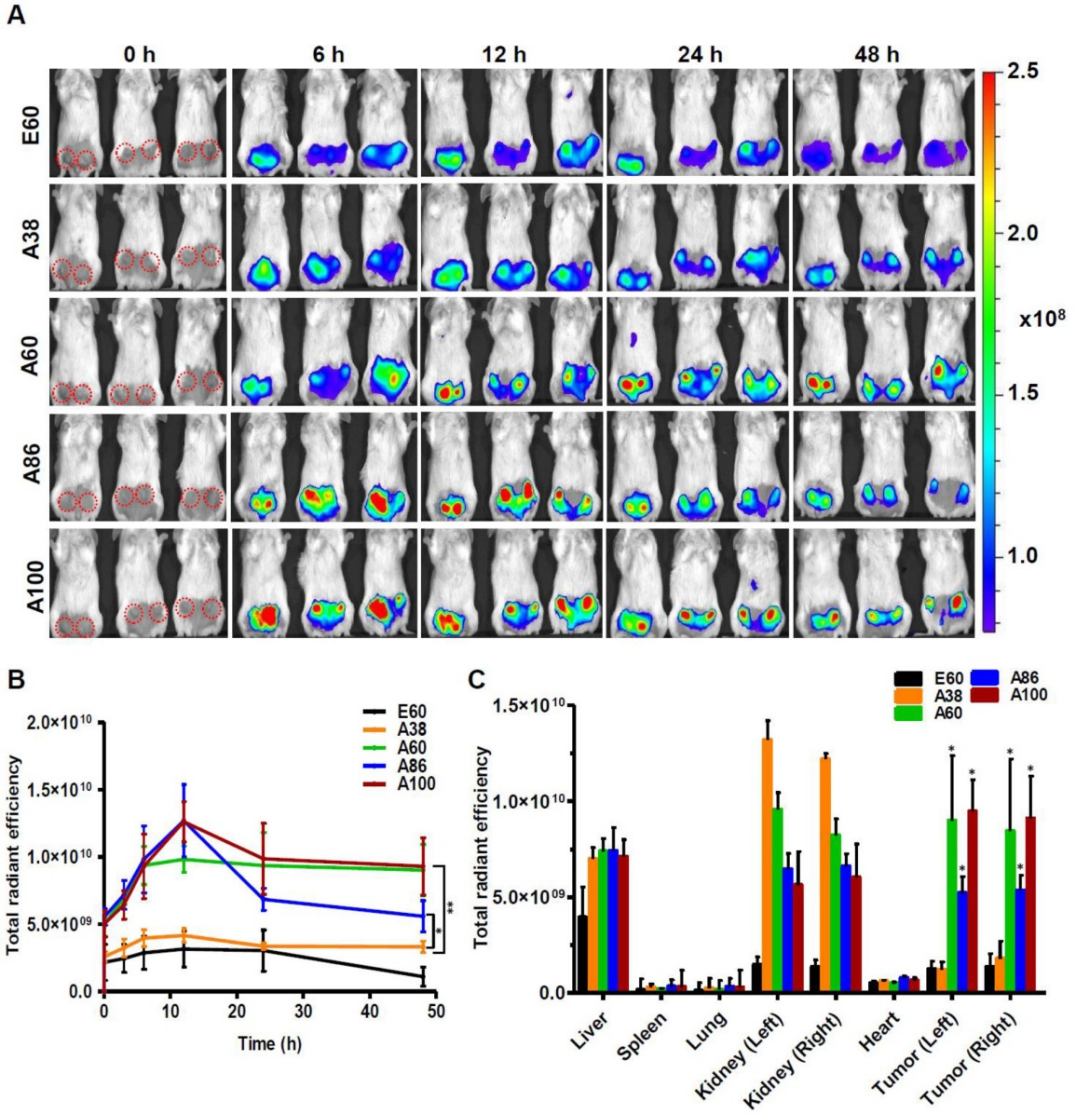
*** In vivo* biodistribution of AP1-ELPs in orthotopic mice model.** (A) FPR 675-labeled E60, A38, A60, A86, and A100 (100 *µ*M) were injected intravenously into 4T1 tumor bearing orthotopic mice after five days of implantation. The representative IVIS images were collected at different time points were shown. (B) The fluorescence intensities in tumor sites was determined at different time points (0, 6, 12, 24, 48 h) respectively. The data presented as mean ± SEM (n=3), the statistically significance were calculated using one-way ANOVA with Bonferroni correction. **P<0.01, *P<0.05 (C) Histogram represents the fluorescence intensities of excised tumors and organs after 48 h post-injection. All Data were represented as mean ± SD (n = 3) and analyzed by one-way ANOVA, *P<0.05.

Similarly, time dependent tumor accumulation studies in 4T1 orthotopic mice model clearly confirmed the gradual accumulation of AP1-ELPs at tumor site (Figure 7A). The lowest molecular weight A38 showed less tumor accumulation than other AP1-ELPs. As seen in 4T1 allograft model, A60 exhibited a rapid increase at tumor sites in a time-dependent manner, with maximum accumulation at 12 h and persisted over 48 h (Figure 7B). Likewise, micelle forming AP1-ELPs (A86 and A100) displayed significant accumulation in tumor tissue with peaked at 12 h. We also observed a gradual decreased in A86 accumulation from the tumor site over time while in the case of A100, the higher signal was retained at tumor site for longer time. Interestingly, the tumor retention of A60 was significantly higher than A86 at 24 h and 48 h (Figure 7B) respectively, suggesting that apart from the molecular weight of polypeptide, other factors such as charge, shape or valence of targeting ligands might influence on tumor retention capacity. Taken together, these studies demonstrated the possible patterns of tumor accumulation by different form of targeted based ELPs in both allograft and orthotopic mice models. Thus, in future studies, the distinctive features of non-micelle or micelle forming AP1-ELPs would be exploited for clinical application in delivering therapeutic drugs like doxorubicin and paclitaxel to target solid tumors.

**Figure 8:**
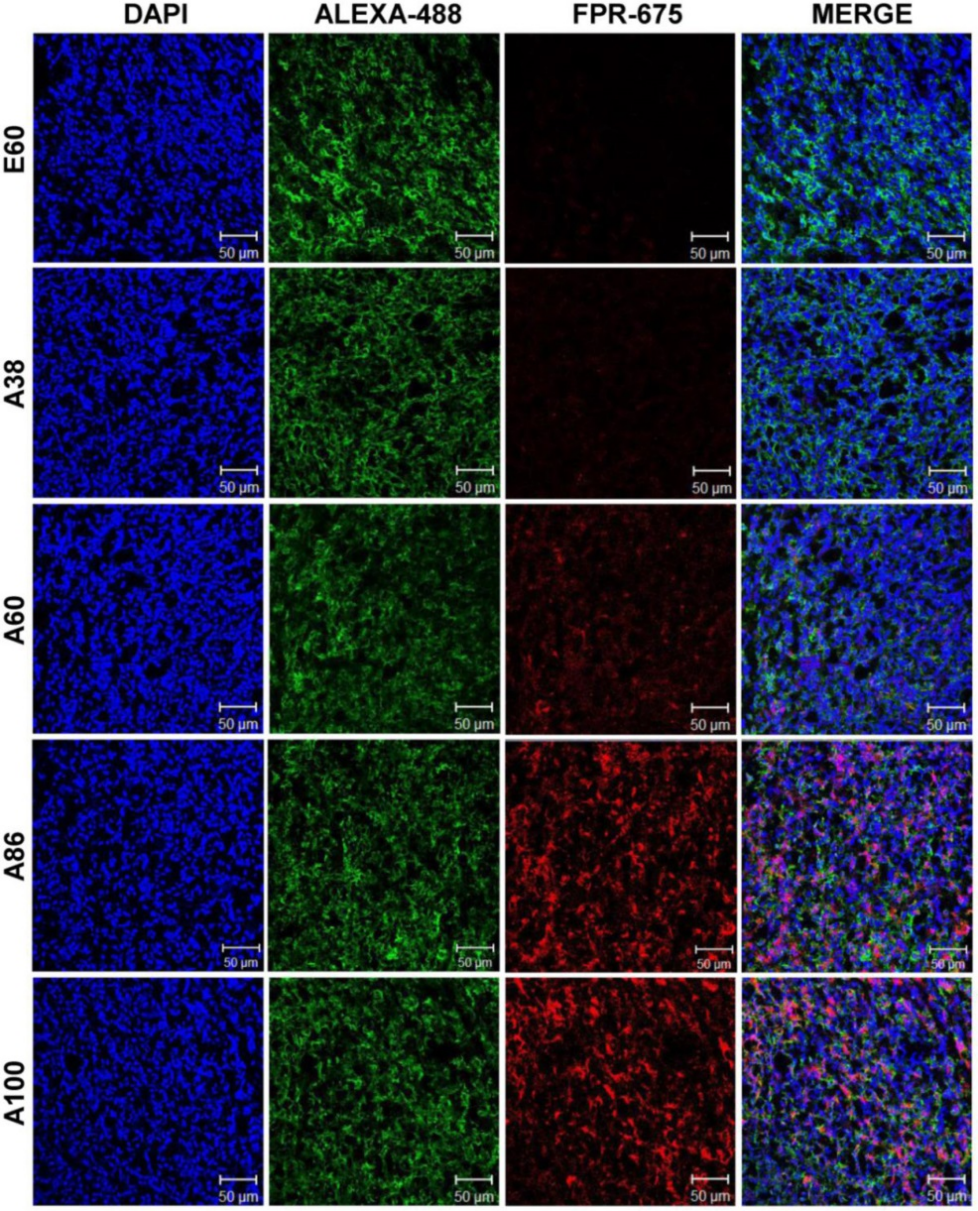
Immunohistology staining of tumor tissues extracted from 4T1 tumor xenograft mice after 48 h post-intravenous injection of respective polypeptides. Tumor tissue sections were stained with anti-IL-4R and observed under a confocal microscope. Representative images of 10 repetitive experiments. Blue, nuclei stained with DAPI; Green, tumor cells stained with anti-IL-4R; Red, labeled polypeptide. Scale bar, 50 µm.

Subsequently, the *ex vivo* fluorescence images of tumors and organs from 4T1 orthotopic mice clearly revealed maximum tumor retention of A60 and A100 compared to other polypeptides (Figure 7C and Figure S14, Supplementary Information). The low molecular weight A38, showed high signal in kidney which may be due to rapid clearance of low molecular weight through kidney filtration. In contrary, the large molecular weight polypeptides A60, A86 and A100 showed slightly higher liver uptake, possibly due to hepatic clearance over time 36. Taken together, in both the tumor models, the high molecular weight AP1-ELPs display better tumor penetration with extensive retention in tumor tissue along with reduced non-specific accumulation in the vital organs. Furthermore, immunohistology examination of tumor tissues obtained from 48 h post-administration of ELPs in 4T1 allograft mice confirmed that A86 and A100 were significantly confined to the tumor tissues even after 48 h, as compared to other polypeptides (Figure 8).

## Conclusion

Taken together, our results collectively verified that a multivalent ELP-based delivery system has excellent tumor-targeting potential. The molecular weight and structural conformation of the polypeptides play vital roles in determining tumor penetration, biodistribution, as well as retention time in tumor tissue. For the first time, we evaluated the biodistribution patterns of micelle-forming and non-micelle-forming targeting ELP polymers. Based on the cellular binding and internalization assay, we confirmed that the micelle forming polypeptides displayed superior cellular accumulation and uptake than non-micelle forming polypeptides. Determination of targeting activity *in vivo* in two different mice model further confirmed that the high molecular weight micelle-forming targeting AP1-ELPs have great potential as carriers, in terms of tumor localization as well as retention. At the same time, these approaches are further optimized for the delivery of various therapeutic drugs for clinical applications. The mechanisms of drug accumulation and release as well as comparison of the therapeutic effects between micelle-forming and non-micelle-forming AP1-ELPs in association with small molecular drugs will be elucidated. As IL-4R is overexpressed in different types of the solid tumor, we believed that these AP1-ELPs would provide a promising platform for targeted therapy of various cancers.

## Experimental Section

### Materials

Synthetic oligonucleotides encoding monomer genes of V_7_, V_3_G_3_A_3_, and AP1-V_5_ were obtained from Macrogen Inc. Seoul. All the restriction enzymes were purchased from New England Biolab. The competent BL21 (DE3) *E. coli* cells and DH5α were procured from Invitrogen, Carlsbad, CA, USA. Circle grow media (MP Biomedicals, CA, USA), 100 μg mL-1 of ampicillin (AMRESCO, LLC, OH, USA) and 1 mM IPTG (Carbosynth Limited, Berkshire, UK) were used for protein expression. Sodium chloride and copper chloride were acquired from Sigma Aldrich, St. Louis, MO, USA. Flamma 675 Vinylsulfone was obtained from BioActs, Incheon, Korea. Sulfo-SMCC (succinimide 4-[N-maleimidomethyl] cyclohexane carboxylate) and other chemicals are acquired from Sigma Aldrich. 4T1 murine breast cancer cells and MDA MB231 human breast cancer cells were obtained from the American Type Culture Collection (ATCC). 4T1 cells were grown in RPMI-1640 (Hyclone), and MDA MB231 cells were grown in Dulbecco's modified Eagle's medium (DMEM) containing 10 % fetal bovine serum (Sigma Aldrich), 100 U/mL of penicillin, and 100 μg/mL of streptomycin (Hyclone). Cells were maintained at 37 °C containing 5% CO_2_.

### ELP Nomenclature

ELPs were designated as ELP [X_a_Y_b_Z_c_]n, where X, Y, Z specify the guest residue, a, b, and c are the numbers of corresponding guest residue repeats, and 'n' denotes the number of monomer gene repeats for RDL. For example, V_3_G_4_A consists of seven pentapeptide XGVPG repeats with Valine, Glycine, and Alanine as guest residues (X). To avoid confusion, we named the ELP and AP1-ELPs according to their molecular weight. The estimated molecular weight of non-targeting (V_3_G_3_A_1_)_16_(V_7_)_5_ was 60 KDa and thus termed as E60. Similarly, targeting polypeptides containing AP1 peptides [(AP1V_5_)V_7_]_6,_ (AP1V_5_)_8_(V_3_G_3_A_1_)_12_, (AP1V_5_)_16_-[(V_3_G_3_A_1_)_3_V_7_]_3_, and (AP1V_5_)_16_(V_3_G_3_A_1_)_12_(V_7_)_5_ were abbreviated as A38, A60, A86, and A100, respectively.

### Thermal Characterization of ELPs

The transition temperatures (Tt) of E60, A38, A60, A86, and A100 were determined by monitoring the turbidity profile of protein solutions as a function of temperature, using a UV-visible spectrophotometer (Agilent Technologies, CA, USA) at 350 nm. Absorbance was monitored at 25 μM concentration, from 20 ºC to 55 ºC with 1ºC/min increments. The first derivative of the turbidity profile with respect to temperature was numerically calculated, and the Tt was defined as the solution temperature at 50% of maximum turbidity.

### Dynamic Light Scattering (DLS)

Particle sizes of E60, A38, A60, A86, and A100 were measured in the temperature range 20°C to 55°C, at increments of 1°C/min, using a Wyatt dynaPro NanoStar (Wyatt technology, Santa Barbara, CA, USA). The size distribution of the hydrodynamic radius was determined by a DLS detector at 90°.

### Cryo-TEM Imaging

For accurate confirmation of particle size, cryo-TEM images were taken using FEI Tecnai transmission electron microscope (Oregon, USA). Filtered protein samples (25 μM) were incubated at 37 °C for 10 min, and a small drop of protein was air-dried on carbon-coated copper grids for 5 min, followed by observation under Cryo-TEM after negative staining with uranyl acetate.

### Circular Dichroism Spectra (CD)

Circular dichroism (CD) spectroscopy was used to investigate the secondary structures of the polypeptides according to temperature. The CD spectra were examined on a Jasco-1500 circular dichroism spectrometer (JASCO, Maryland). Protein samples were diluted to 25 μM in PBS and filtered through a 0.2-*μ*m Whatman syringe filter (GE Healthcare, Amersham, UK) to remove impurities. The CD spectra were measured at the different temperatures of 20, 25, 30, 35, 40, 50, and 60 °C in a 0.1-cm path length cell. The spectra were recorded from 180 to 260 nm at a scan speed of 50 nm/min.

### Flow Cytometry

4T1 and MDA MB231 cells were used to observe the binding activities of the respective polypeptides. A total of 1 x 10^6^ cells was incubated with 0.3125 μM Alexa 488-labeled polypeptides for 1 h at 4 °C and 37 °C. The cells were further washed three times with PBS and suspended in 300 μL of PBS. The percentages of binding were analyzed by flow cytometry (BD Bioscience, San Jose, CA, USA). Ten thousand events were collected for each sample for the analysis.

### Confocal Microscopy

4T1 and MDA MB231 (1 x 10^5^) cells were seeded on a 4-well chambered slide and grown to 80% confluence. Cells were then incubated with 0.3125 μM Alexa 488-labeled polypeptides for 1 h at 4 °C and 37 °C. Cells were washed with PBS and fixed with 4 % paraformaldehyde (Sigma Aldrich). Cells were immediately observed using a Zeiss LSM-510 Meta confocal microscope after nuclei and plasma membranes were stained with Hoechst and Wheat Germ Agglutinin for 10 min.

### Lysosome and Early Endosome Staining

MDA MB231 cells were seeded on a cover glass inside a 6-well chambered dish filled with appropriate culture media. After 24 h, cells were incubated with Alexa-488-labeled respective ELPs (0.3125 μM) for 1 h at 37 °C. Cells were washed with PBS and further incubated with pre-warmed culture medium containing lysotracker deep red (Molecular Probes, Life Technologies Corporation, Eugene, OR, USA). Later, the cells were fixed with 4 % paraformaldehyde, nuclei-stained with Hoechst, and observed under a confocal microscope. For early endosome staining, cells were seeded on a 4-well chambered slide to 80% confluence. Cells were then treated with respective ELPs for 30 min or 1 h. Early endosomes were stained by anti-EEA1 (BD Biosciences, Franklin Lakes, NJ, USA) for 1 h at 37 °C, followed by Alexa Flour-594 goat anti-mouse IgG (Life Technologies Corporation, Eugene, OR, USA) secondary antibody for 1 h. The cells were observed under a confocal microscope after nuclei were stained with Hoechst.

### *In vivo* Fluorescent Imaging

All animal experiments were conducted according to the guidelines of the Animal Care and Use Committee of Kyungpook National University (Permit Number KNU 2018-0017). Athymic wild-type mice (BALB/c) were housed in a specific pathogen-free environment. Tumors were created by subcutaneously injecting 4T1 cells (5 ×10^6^ cells) into the right flanks of 6-week-old female mice to generate allograft mice. For orthotopic mice model, 5×10^5^ of 4T1 cells were implanted into left and right mammary fat pad and allow to grow for 5 days before imaging. The length (a) and width (b) of each tumor were measured using a caliper in order to calculate tumor volume (mm^3^) by following formula. V=ab^2^/2; a=length, b= width

When the tumors reached nearly 200 mm^3^ in volume, the mice were administered 100 µM FPR-675-labeled E60, A38, A60, A86, and A100 intravenously (n=10). *In vivo* biodistribution was monitored at various time intervals of 10 min, 1, 2, 4, 6, 12, 24, and 48 h post-injection by fluorescence imaging (FLI) using an IVIS *In vivo* Imaging system (Perkin Elmer, USA). Grayscale photographic images and fluorescence images were superimposed using LIVINGIMAGE (version 2.12, Perkin Elmer) and IGOR Image Analysis FX software (WaveMetrics, Lake Oswego, OR). Fluorescence images were obtained at suitable wavelength and spectral unmixing (using LIVINGIMAGE software) were done to remove the nonspecific FLI signals. Fluorescent signals (FLI) were expressed in units of total radiant efficiency. All mice were anesthetized using 1% to 2% isoflurane gas during imaging.

### *Ex vivo* Imaging and Immunohistochemistry

To analyze *ex vivo* organ distribution, animals were euthanized at 48 h post-injection with CO_2_. All the major organs (liver, kidneys, spleen, heart, and lungs) along with the tumor tissues were isolated, and *ex vivo* fluorescence images were taken (*n* = 10). Tumor tissues were further fixed with 4 % paraformaldehyde overnight and rapidly frozen. Tissue slices (8-*μ*m thick) were prepared using a cryo-microtome and stained with anti-IL-4R antibody (R&D Systems; 1:100), followed by Alexa 488-labeled goat anti-mouse IgG secondary antibody (1:200). Further tumor accumulation of respective polypeptides was observed under a confocal microscope after nuclei were stained with DAPI.

### Statistical Analysis

The statistical significance was determined using one-way ANOVA for comparison of multiple groups followed by a Tukey post-hoc test. All data analysis was carried out by Graph pad prism 6.0 software. ***P<0.001, **P<0.01, and *P<0.05 were considered as statistically significant and indicated by asterisks in the figures.
