# Supplementary material for: Effects of molecular weight and structural conformation of multivalent-based elastin-like polypeptides on tumor accumulation and tissue biodistribution
Source: Nanotheranostics. 2020 Jan 15;4(2):57–70. doi: 10.7150/ntno.39804 (PMC7064738; doi:10.7150/ntno.39804)
Supplement: Supplementary file 1 — Supplementary figures and tables. [file ntnov04p0057s1.pdf]

Supplementary materials (Submission no. 39804f2)

Effects of molecular weight and structural conformation of multivalent-based elastin-like polypeptides on tumor accumulation and tissue biodistribution

*Vijaya Sarangthem [1, 2, ‡], Bo-Yeon Seo [1, ‡], Aena Yi [1, ‡], Young-Jin Lee [1], Sun-Ha Cheon [1], Sang Kyoon Kim [4], Thoudam Debraj Singh [3], Byung-Heon Lee [1], Rang-Woon Park [1]**

[1]Department of Biochemistry and Cell Biology, School of Medicine, and Cell & Matrix Research Institute, Kyungpook National University, Daegu 41944, Republic of Korea.

[2]Department of Pathology, All India Institute of Medical Sciences, New Delhi-110029, India.

[3]Department of Medical Oncology Lab., All India Institute of Medical Sciences, New Delhi-110029, India.

[4]Laboratory Animal Center, Daegu-Gyeongbuk Medical Innovation Foundation, Cheombok, Daegu, 41061, Republic of Korea

[‡]Vijaya Sarangthem, Bo-Yeon Seo and Aena Yi equally contributed to this work

Corresponding author:

*E-mail: (R-W Park) nwpark@knu.ac.kr, phone No.: +82 53 420 4822,

Fax No. : +82 53 422 1466

METHODS

Preparation of AP1-ELPs and ELP

Synthetic oligonucleotides encoding monomer genes of V_7, $V_3G_3A_3$, and AP1-V_5 containing *Bam*H I, *Pfl*M I, *Bgl* I, and *Hin*D III enzyme sites were obtained from Macrogen Inc. Seoul, Korea. The corresponding oligonucleotides were annealed and ligated into *Bam*H I and *Hin*D III doubled-digested pRSET B vector. Each gene was used as a monomer gene to synthesize ELPs or AP1-ELPs with variable lengths by RDL. Later, AP1-ELP polypeptides of different lengths were ligated with different ELPs of variable sequence in order to generate polypeptides with diverse molecular weights. Briefly, non-targeting ELP control was generated by ligating $(V_3G_3A_1)_{16}$ with $(V_7)_5$ abbreviated as E60. Likewise, targeting AP1-ELPs were designed by varying ELP sequences together with number of targeting peptides (AP1). All the targeted polypeptides were constructed to have transition temperatures (Tt) between 37-42 °C. All genes were confirmed through gene sequencing (Macrogen Inc. Seoul, Korea).

Expression and purification of AP1-ELPs and ELP

For protein expression, respective genes were transformed to BL21 (DE3) competent cell *E. coli*. Transformed *E. coli* were cultured in Circle grow media, and protein expression was induced by IPTG induction for 4 h. After 4 h, *E. coli* were harvested and subjected to protein purification using the inverse transition cycling (ITC) method. Three rounds of ITC with NaCl were performed to remove cell debris. An additional three rounds of ITC after incubation at 45 °C were performed for high molecular weight polypeptides such as A86 and A100. Final proteins were suspended in PBS and stored at -20 °C.

Mass spectrometry analysis

The accurate molecular weight of respective polypeptides were assessed using an MALDI TOF/TOF MS (4700 Proteomics Analyser, Applied Biosystems, Farmingham, MA, USA).

For the measurement, polypeptides were suspended with 0.1% trifluoroacetic acid and mixed with an equal volume of matrix solution (1:1). Resultant mixture (1 µL) was then applied to a standard steel target for drying at room temperature. The spectra were obtained after calibration with standards.

Fluorescent labeling of ELPs

All polypeptides were diluted in PBS at a final concentration of 100 µM and mixed with Sulfo-SMCC (succinimide 4-[N-maleimidomethyl] cyclohexane carboxylate) (Sigma Aldrich) at an equivalent molar concentration. Alexa Fluor 488-C5 maleimide (Invitrogen) dye dissolved in DMSO was added to the mixture at the same molar concentration as the protein. The reaction was maintained by constant rotation at 4 ºC overnight. Unexpected products were eliminated by dialysis (MWCO 6-8,000) for 24 h and filtered through Ultracel ®-30K centrifugal filters (Merck Millipore Ltd., Ireland). Degree of labeling was determined according to the Invitrogen conjugation manual.

For *in vivo* experiments, Flamma 675 Vinylsulfone (BioActs, Incheon, Korea) was suspended in DMSO and added dropwise to the ELP solution at a 1:2 (protein : dye) ratio. The reaction was allowed to proceed at 4 ℃ overnight with constant rotating. The labeled proteins were purified by G-25 column chromatography (GE Healthcare, UK) to eliminate free dye and concentrated through Ultracel®-30K centrifugal filters. The labeled protein efficiency was assessed by UV–vis spectrophotometry (Agilent Technologies, CA, USA) according to the company manual and stored at -20 ºC.

Stability of ELPs in plasma

The stability of the ELPs were checked by incubating all polypeptides (50 µM concentration) with 100% plasma at 37°C. After 24 h incubation all the proteins were subjected to SDS-PAGE analysis after 6 times dilution with PBS. The stability of the ELPs in plasma were

further confirmed by measuring the size through Dynamic light scattering (DLS). In order to avoid the hindrances from lipoproteins and other particles present in plasma during observation, ELP proteins post plasma incubation were precipitated out with 2M NaCl. The ELPs particle size were then measured after dissolving the precipitate with cold PBS.

Pharmacokinetics analysis

4T1 tumor-bearing mice (100 mm^3) were injected with FPR 675-labeled E60, A38, A60, A86, and A100 (100 μM) intravenously (n = 4 for each group). At various time intervals (10 min, 20 min, 30 min, 1 h, 2 h, 4 h, 6 h, 24 h, and 48 h), 20 μL of blood was sampled out from the tail vein, collected in heparinized tubes, and further centrifuged at 13,000 ×g for 5 min to collect plasma. Fluorescence intensity present in plasma was measured using a SpectraMax M plate reader. Plasma fluorescence intensity of respective polypeptides were calculated from the standard curve generated using known concentrations of labeled proteins (0.625-20 μM). Pharmacokinetic parameters were determined by Non-compartmental analysis.

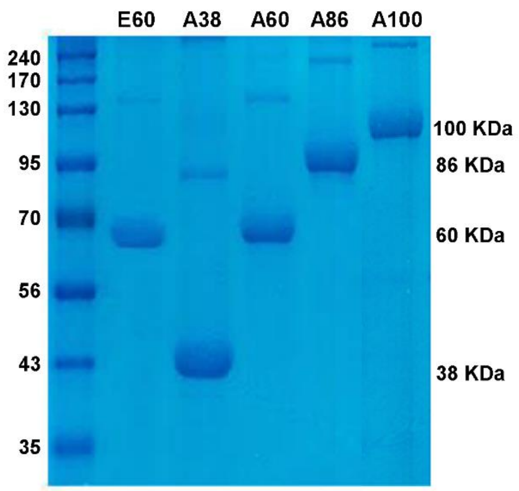

Figure S1. SDS–PAGE analysis of E60, A38, A60, A86, and A100 stained with copper chloride. From left, lane 1: Pre-stained protein marker; lane 2: E60 (~60 KDa); lane 3: A38 (~38.9 KDa) lane 4: A60 (~60 KDa); lane 5: A86 (~86 KDa); lane 6: A100 (~100 KDa). Upper band indicates existence of dimer due to the presence of cysteine residue.

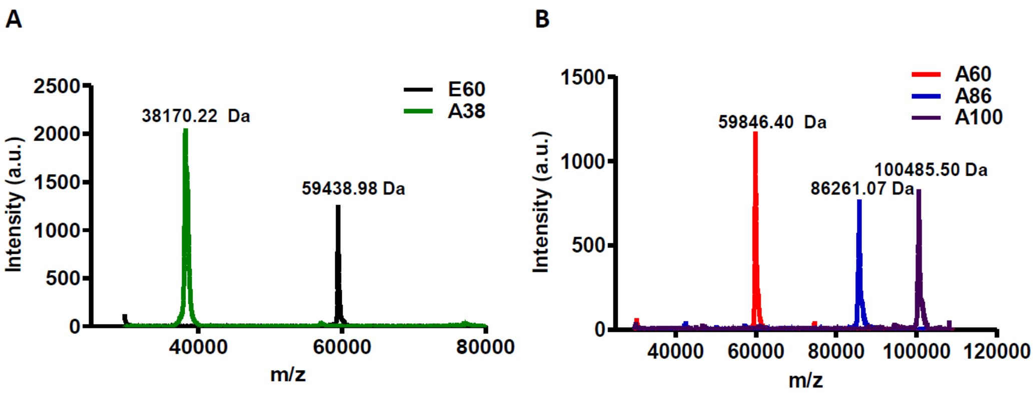

Figure S2. (A-B) MALDI-TOF MS spectra of E60, A38, A60, A86, and A100. Respective molecular weights (Dalton) are indicated near the peaks.

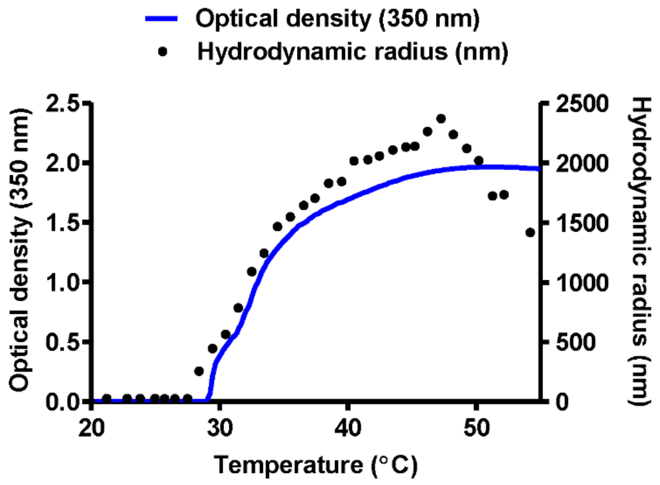

Figure S3. Hydrodynamic radius (nm) and turbidity profiles of E60 monitored at different temperatures (1 °C/min). Absorbance was monitored from 20 ºC to 55 ºC at 25 μM concentration.

A86

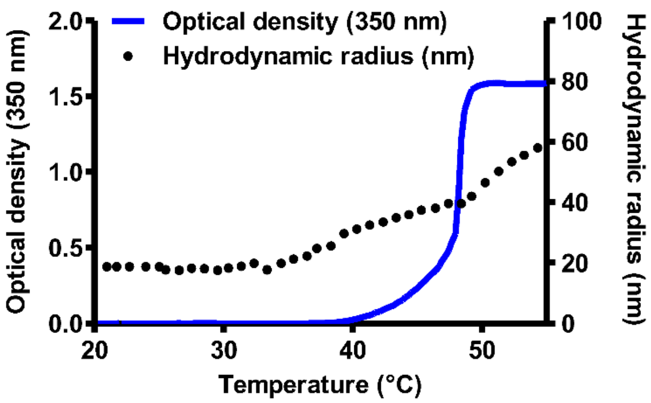

A100

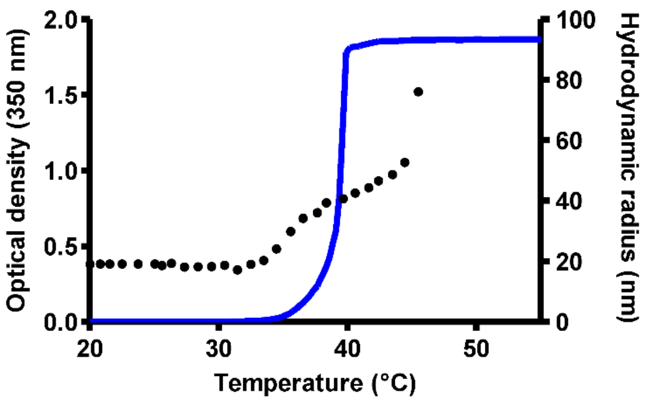

Figure S4. Turbidity profile and hydrodynamic radius of A86 and A100 measured at different temperature at the rate of 1°C/ min increment. Absorbance was monitored from 20 ºC to 55 ºC at 25 μM concentration.

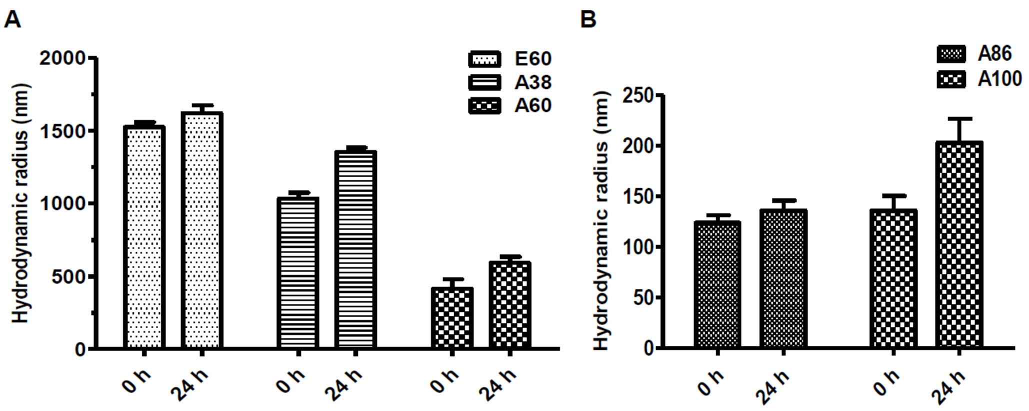

Figure S5. The stability of the ELPs in plasma were further checked by measuring the size through Dynamic light scattering (DLS). The hydrodynamic radius of the ELP at 0 h and 24 h post-incubation with 100% plasma at 37°C were determined. Data are represented as mean ± SD (n = 3).

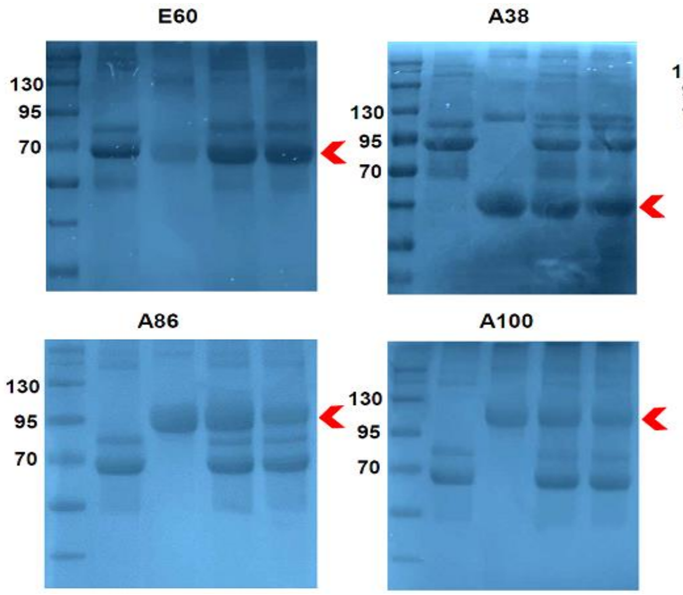

Figure S6. The stability of the ELPs were checked by incubating all polypeptides of 50 μM concentration with 100% plasma at 37°C for 24 h. Then all the polypeptides were subjected to SDS-PAGE analysis (after 6 times dilution with PBS) to further confirmed the stability of all the polypeptides in plasma. Red arrow indicated respective ELPs. Lane 1; protein ladder (KDa), Lane 2; plasma only, Lane 3; ELPs diluted in PBS, Lane 4; ELPs in plasma incubated 0 h and Lane 5; ELPs in plasma incubated 24 h.

A

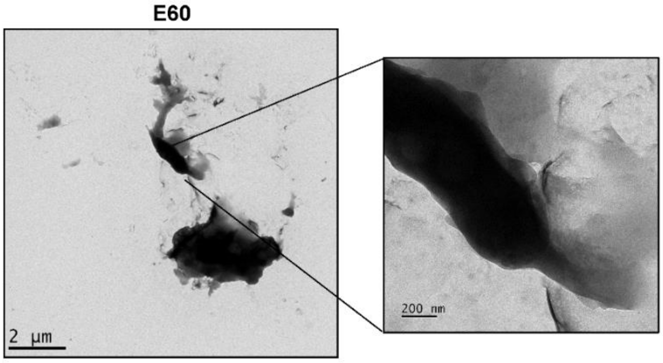

B

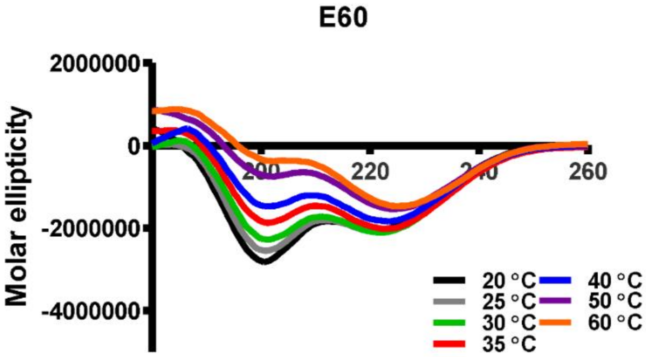

Figure S7. (A) TEM images of E60 obtained at 37 °C. Scale bars indicate 2 µm or 200 nm. (B) Circular dichroism spectra of E60 were taken at different temperatures (20, 25, 30, 35, 40, 50, and 60 °C).

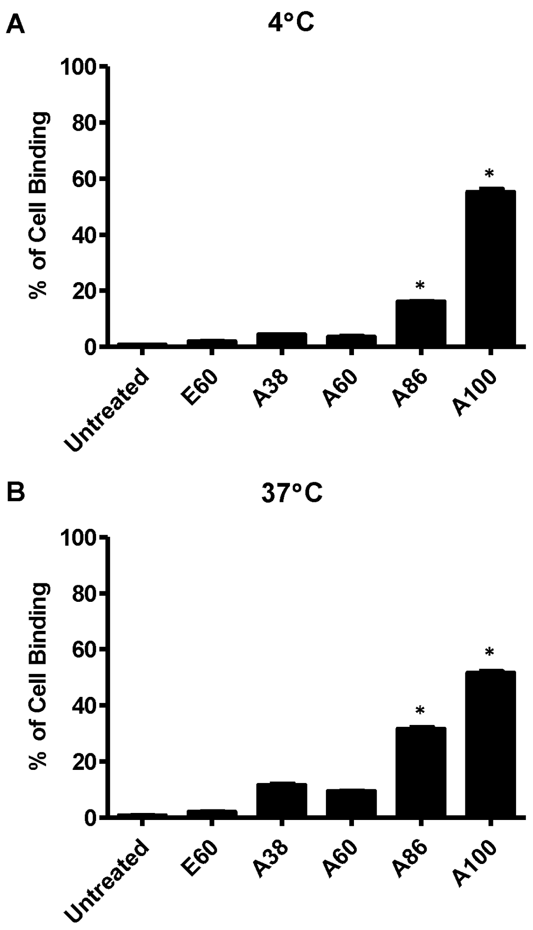

Figure S8. 4T1 cells were treated with Alexa-488-labeled E60, A38, A60, A86, and A100 (0.3125 µM) for 1 h at 4 °C (A) and 37 °C (B), and percentages of binding were determined using flow cytometry. Data are represented as the mean ± s.d. (n = 6). **P*<0.05, statistical significance was determined by one-way ANOVA analysis.

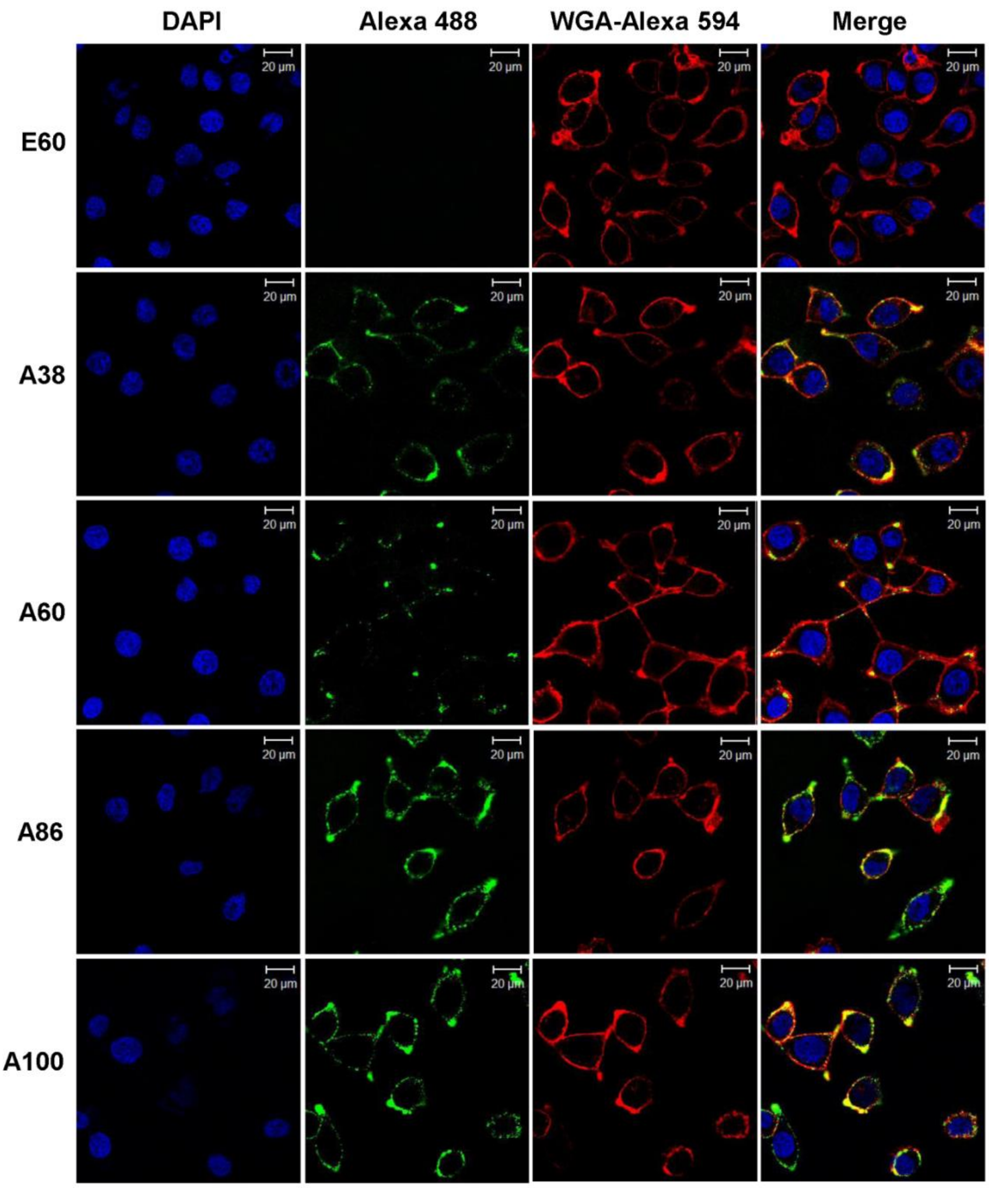

Figure S9. Cellular accumulation of AP1-ELP polypeptides: MDA MB231 cells were treated with Alexa-488-labeled respective polypeptides (0.3125 μM) for 1 h at 4 °C. After cells were fixed with 4 % paraformaldehyde, cell membranes and nuclei were stained with WGA Alexa 594 and Hoechst. Representative confocal images of three experiments, (scale bar, 20 μm).

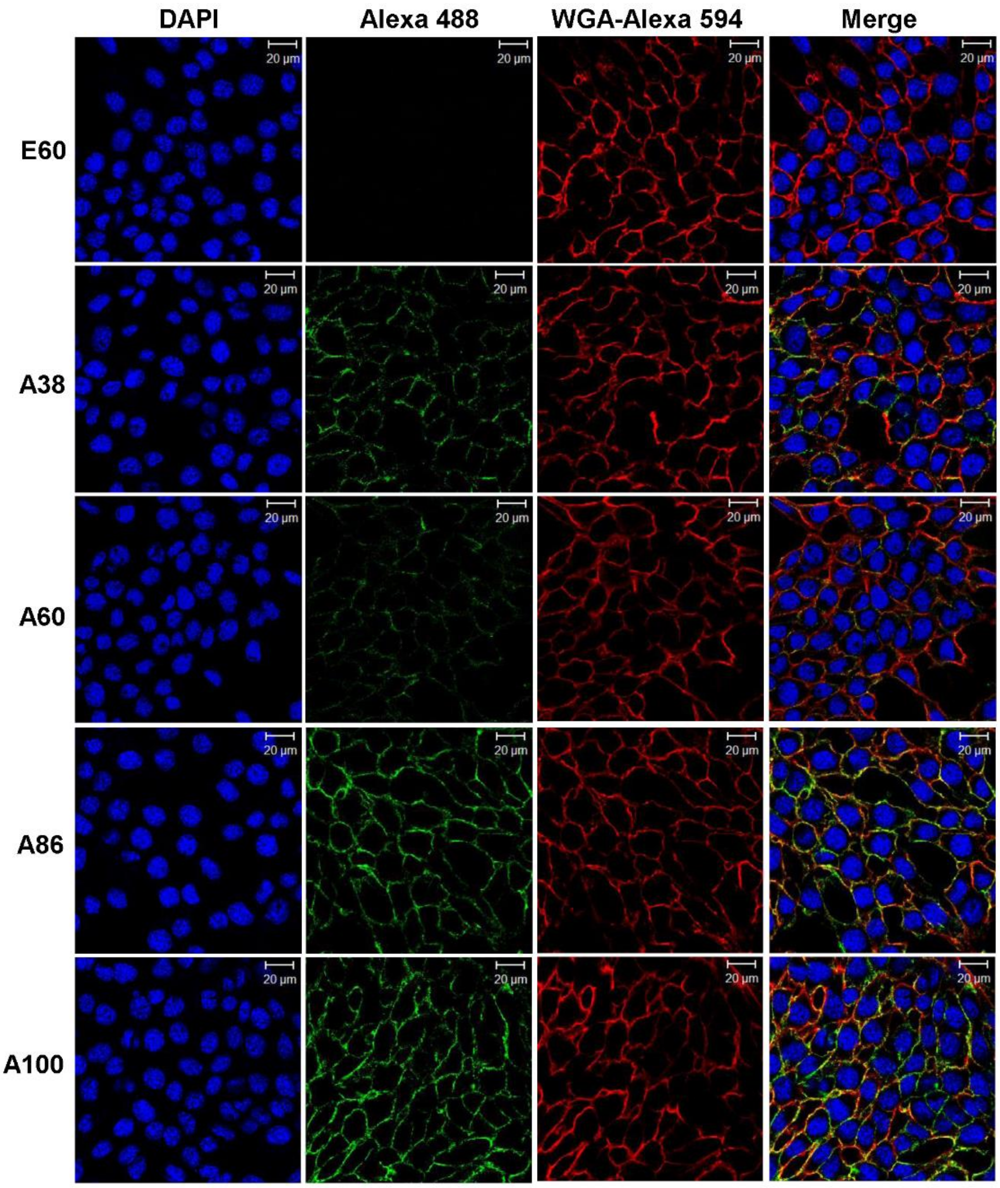

Figure S10. Confocal images of 4T1 cells treated with respective polypeptides at 4 °C. Cell membranes and nuclei were stained with WGA Alexa 594 and Hoechst. Representative confocal images of three experiments (scale bar, 20 μm).

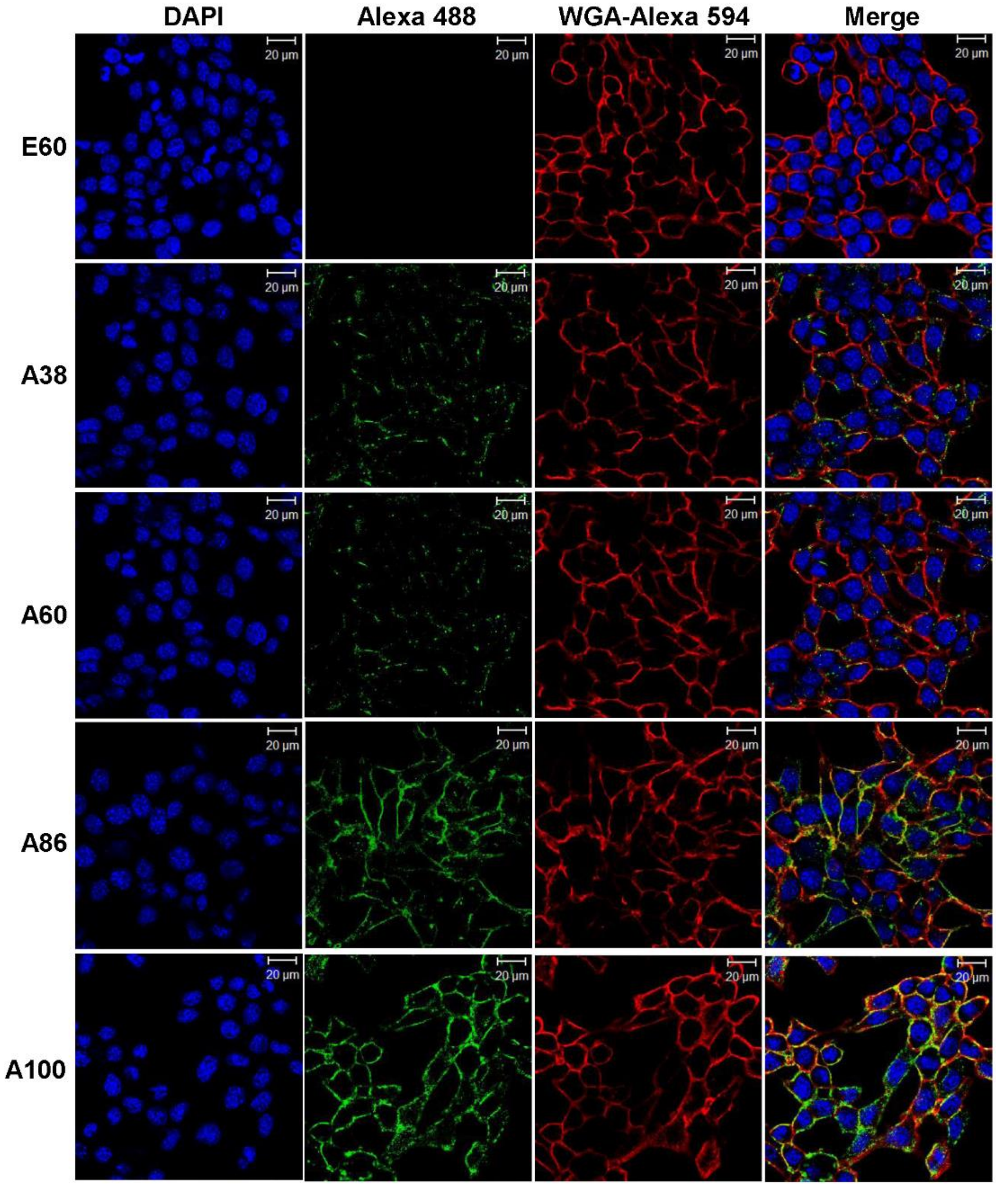

Figure S11. Confocal images of 4T1 cells treated with respective polypeptides at 37 °C. Cell membranes and nuclei were stained with WGA Alexa 594 and Hoechst. Representative confocal images of three experiments (scale bar, 20 μm).

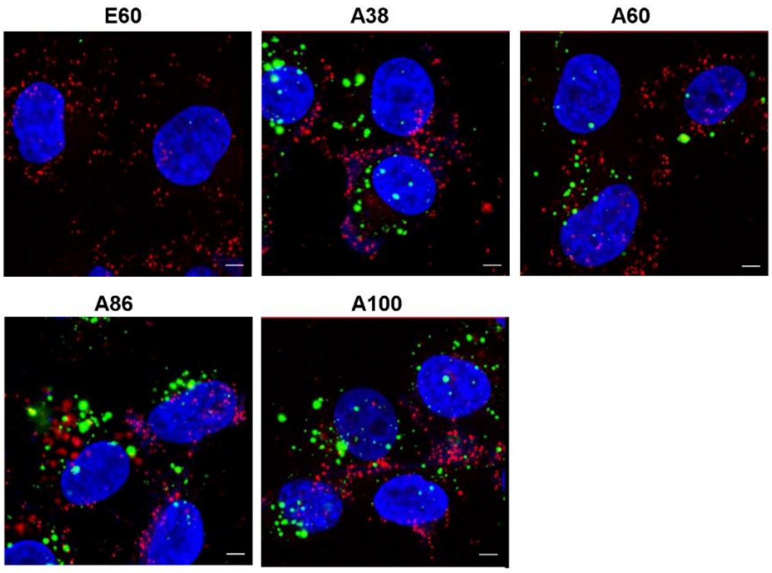

Figure S12. MDA MB231 cells were treated with Alexa-488-labeled respective polypeptides (0.3125 μM) for 1 h at 37 °C. After cells were fixed with 4 % paraformaldehyde, early endosomes were stained with anti-EEA1 for 1 h at 37 °C. Cells were observed under a microscope after nuclei were stained with Hoechst. *Right panels*: Co-localization of polypeptides with early endosome observed by Z-section scanning of confocal microscopic images. Representative confocal images of three experiments. Hoechst: nuclear stained with blue, Red: early endosome (EEA1), Green: polypeptides labeled with Alexa-488, (Scale bar, 10 µm).

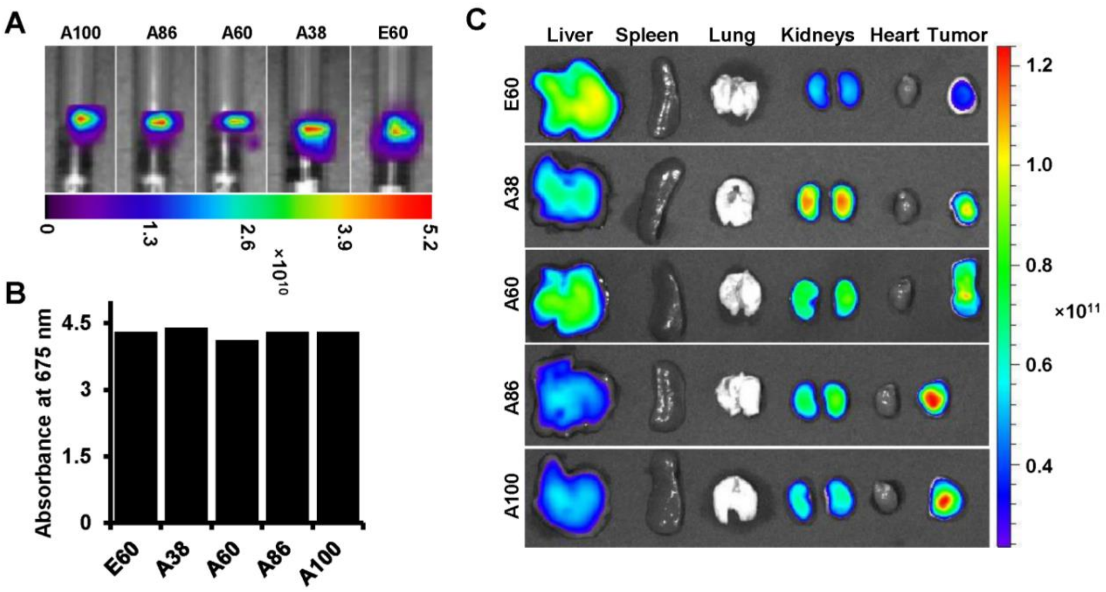

Figure S13. Biodistribution of AP1-ELP polypeptides. (A) FPR 675-labeled E60, A38, A60, A86, and A100 were analyzed before injection to confirm the fluorescence intensities of the polypeptides. (B) Quantification of fluorescence intensities measured at an excitation wavelength at 675 nm. (C) FPR 675-labeled polypeptides were injected intravenously into 4T1-tumor allograft mice, and organs were extracted at 48 h post-injection. IVIS images of excised tumors and organs (n=10).

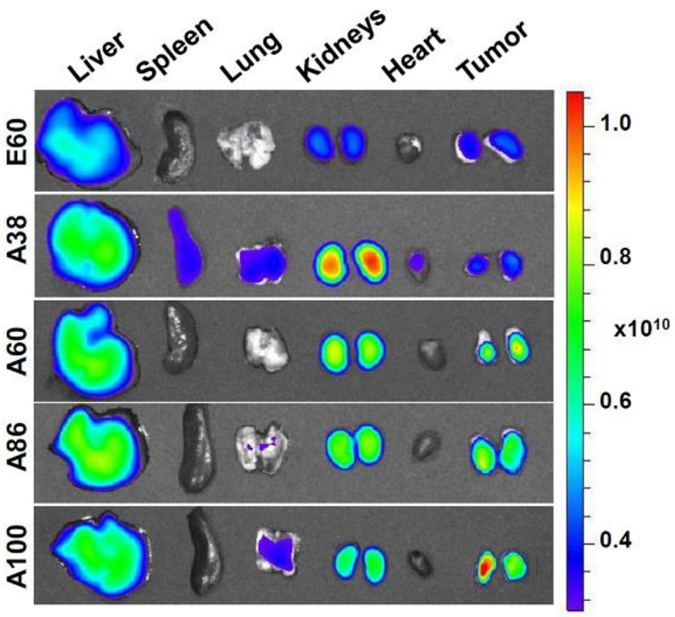

Figure S14. For *ex vivo* imaging, all vital organs including tumor tissue were extracted from 4T1-tumor orthotopic mice, after 48 h intravenous injection of FPR 675-labeled polypeptides. Representative IVIS image of excised tumors and organs (n=3).

Table S1. Secondary structural components ratio obtained from measured CD spectra (residue molar ellipticity) of AP1-ELPs at various temperatures.

Samples	Structures	20°C	25°C	30°C	35°C	40°C	50°C	60°C
E60	Helix	14.6	16.0	17.3	17.8	15.9	13.8	14.1
	Beta	9.5	8.5	8.2	11.5	21.5	35.7	39.5
	Turn	24.4	25.1	25.7	25.1	21.5	16.9	16.0
	Random	51.6	50.3	48.7	45.6	41.1	33.6	30.5
A38	Helix	13.4	15.6	16.8	18.4	19.1	22.0	24.8
	Beta	0	0	0	0	0	0	0
	Turn	15.8	16.4	17.3	20.8	21.2	23.2	24.2
	Random	70.8	68.0	65.9	60.8	59.7	54.8	51.0
A60	Helix	24.2	24.9	25.4	25.9	26.2	25.7	26.4
	Beta	0	0	0	0	0	0	0
	Turn	27.4	27.3	27.5	27.5	28.2	29.7	30.8
	Random	48.5	47.8	47.0	46.6	45.6	44.7	42.9
A86	Helix	37.3	27.9	28.5	28.7	29.3	22.0	16.3
	Beta	0	0	0	0	0	16.6	35.4
	Turn	30.1	30.0	30.0	30.2	30.5	26.3	18.2
	Random	42.6	24.1	41.5	41.1	40.2	35.0	30.1
A100	Helix	26.7	27.0	27.6	28.2	18.6	10.7	11.6
	Beta	0	0	0	0	21.9	47.9	47.2
	Turn	31.2	31.3	31.4	31.8	23.6	12.4	12.9
	Random	42.1	41.7	41.0	40.0	35.9	29.0	28.3

Table S2. Plasma pharmacokinetic parameters of AP1-ELP polypeptides. Plasma clearance of labeled polypeptides were analyzed using a Non-compartmental pharmacokinetic model. Data are represented as mean ± SD (n = 4).

Parameters	E60	A38	A60	A86	A100
$T_{1/2}$ (h)	25.5±3.1	60.7±5.6	68.6±4.9	64.5±8.5	63.6±11.1
C_{max} (μg/ml)	6000	3800	6000	8600	10000
AUC (h*μg/ml)	130086.6±15.7	88124.1±7.2	143761.3±13.2	193658.2±22.8	229498.9±36.9
V_d (L/kg)	0.05820	0.07517	0.07391	0.07618	0.07521
Cl (L/hr/kg)	0.00149	0.00079	0.00074	0.00081	0.00088

($T_{1/2}$: terminal half-life; C_{max}: maximum plasma protein concentration; AUC: area under plasma concentration time curve; V_d: volume of distribution; Cl: plasma clearance. Values represented as mean ± s.d., *n* = 4).
